# Single‐cell landscape revealed immune characteristics associated with disease phases in brucellosis patients

**DOI:** 10.1002/imt2.226

**Published:** 2024-07-23

**Authors:** Yi Wang, Siyuan Yang, Bing Han, Xiufang Du, Huali Sun, Yufeng Du, Yinli Liu, Panpan Lu, Jinyu Di, Laurence Don Wai Luu, Xiao Lv, Songnian Hu, Linghang Wang, Rongmeng Jiang

**Affiliations:** ^1^ Experimental Research Center, Capital Institute of Pediatrics Beijing China; ^2^ Beijing Key Laboratory of Emerging Infectious Diseases, Institute of Infectious Diseases, Beijing Ditan Hospital Capital Medical University Beijing China; ^3^ Beijing Institute of Infectious Diseases Beijing China; ^4^ National Center for Infectious Diseases, Beijing Ditan Hospital Capital Medical University Beijing China; ^5^ National Key Laboratory of Intelligent Tracking and Forecasting for Infectious Diseases Beijing China; ^6^ Clinical and Research Center of Infectious Diseases, Beijing Ditan Hospital Capital Medical University Beijing China; ^7^ Beijing Quality Control and Improvement Center of Infectious Disease Beijing China; ^8^ The Department of Infectious Diseases The Third People's Hospital of Linfen City Linfen Shanxi China; ^9^ Department of Infectious Diseases The Affiliated Hospital of Qingdao University Qingdao Shandong China; ^10^ Department of Clinical Laboratory The Third People's Hospital of Lifen City Linfen Shanxi China; ^11^ School of Life Sciences University of Technology Sydney Sydney Australia; ^12^ State Key Laboratory of Microbial Resources, Institute of Microbiology Chinese Academy of Sciences Beijing China; ^13^ University of Chinese Academy of Sciences Beijing China

**Keywords:** *Brucella* infection, brucellosis, cytokine storm, immune response, single‐cell sequencing

## Abstract

A comprehensive immune landscape for *Brucella* infection is crucial for developing new treatments for brucellosis. Here, we utilized single‐cell RNA sequencing (scRNA‐seq) of 290,369 cells from 35 individuals, including 29 brucellosis patients from acute (*n* = 10), sub‐acute (*n* = 9), and chronic (*n* = 10) phases as well as six healthy donors. Enzyme‐linked immunosorbent assays were applied for validation within this cohort. *Brucella* infection caused a significant change in the composition of peripheral immune cells and inflammation was a key feature of brucellosis. Acute patients are characterized by potential cytokine storms resulting from systemic upregulation of *S100A8*/*A9*, primarily due to classical monocytes. Cytokine storm may be mediated by activating S100A8/A9‐TLR4‐MyD88 signaling pathway. Moreover, monocytic myeloid‐derived suppressor cells were the probable contributors to immune paralysis in acute patients. Chronic patients are characterized by a dysregulated Th1 response, marked by reduced expression of IFN‐γ and Th1 signatures as well as a high exhausted state. Additionally, *Brucella* infection can suppress apoptosis in myeloid cells (e.g., mDCs, classical monocytes), inhibit antigen presentation in professional antigen‐presenting cells (APCs; e.g., mDC) and nonprofessional APCs (e.g., monocytes), and induce exhaustion in CD8^+^ T/NK cells, potentially resulting in the establishment of chronic infection. Overall, our study systemically deciphered the coordinated immune responses of *Brucella* at different phases of the infection, which facilitated a full understanding of the immunopathogenesis of brucellosis and may aid the development of new effective therapeutic strategies, especially for those with chronic infection.

## Highlights

- scRNA‐seq analysis reveals distinct immune responses across the acute, sub‐acute, and chronic phases of *Brucella* infection.
- Acute infection is characterized by a cytokine storm, potentially mediated by the activation of the S100A8/A9‐TLR4‐MyD88 pathway, along with immune suppression driven by Mono‐MDSCs
- Chronic infection is marked by a dysregulated Th1 immune response and widespread exhaustion of T and NK cells.

## Graphical abstract

Single‐cell RNA sequencing of 290,369 cells from 29 brucellosis patients and 6 healthy donors reveals distinct immune profiles across acute, subacute, and chronic phases of the disease. Acute patients experience a cytokine storm triggered by classical monocytes, potentially mediated by the S100A8/A9‐TLR4‐MyD88 signaling pathway. Chronic patients exhibit a dysregulated Th1 response, characterized by reduced expression of IFN‐γ and Th1‐associated gene signatures, alongside T and NK cell exhaustion. This investigation sheds light on the complex immunopathogenesis of brucellosis, providing valuable insights that may facilitate the development of novel therapeutic strategies, particularly for managing chronic infections.

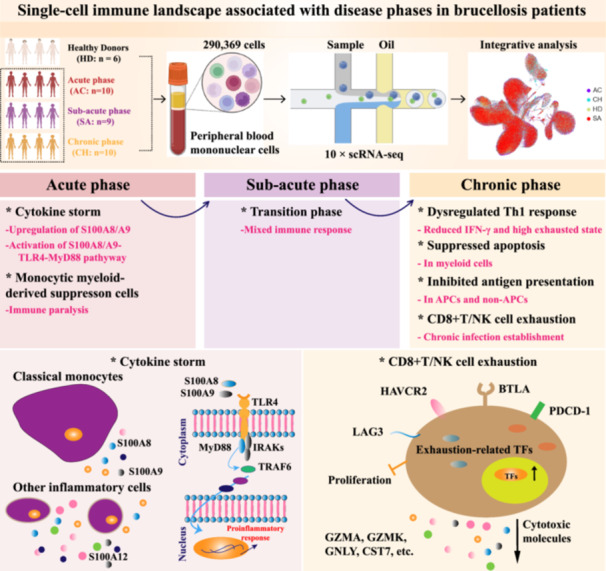

## INTRODUCTION

Brucellosis (also known as Malta fever) is caused by members of the *Brucellae* genus with different species varying in their affinity and virulence in different hosts. The disease affects livestock worldwide and is one of the most important zoonotic diseases in humans [1]. Brucellosis results in human morbidity, and economic losses, and perpetuates poverty. Although extensive efforts have been made to control the spread of brucellosis, it remains endemic in many regions of the world. The incidence of human brucellosis remains very high in endemic regions with >500,000 new infections estimated annually [2]. In Eastern Asia, China has the highest disease burden of brucellosis, with the incidence gradually increasing (from 0.0281/100,000 in 1993 to 5.0553/100,000 in 2021) and geography continuously expanding [3]. Hence, brucellosis remains an important disease that cannot be ignored and continuously contributes to significant health, veterinarian, and economic concerns.

*Brucella* is a facultative intracellular bacterium. It contains several virulence factors for invasion and evading host immunity (e.g., type IV secretion system) [4]. The bacteria can replicate inside phagocytes, (e.g., macrophages and dendritic cells), enabling them to survive, evade and modulate the immune responses [5]. This intracellular lifestyle limits exposure to the host's adaptive and innate immune responses [6]. Although brucellosis is rarely fatal, it is a severe and debilitating chronic illness with prolonged antibiotic dual therapy treatment. Importantly, there are currently no approved human vaccines currently against brucellosis. Hence, it is crucial to understand the disease mechanisms to control brucellosis.

Brucellosis patients present with a broad range of clinical manifestations from asymptomatic to mild/moderate disease, some patients can progress to severe disease involving multiple organs or even death [7]. Brucellosis disease can be classified into three phases based on the severity and duration of symptoms (National Health Commission of China, WS 269‐2019) [8]: acute (initial 3 months), sub‐acute (3–6 months), and chronic stages (more than 6 months) [9]. In the acute stage, brucellosis patients often display nonspecific and variable manifestations, including fever, sweating, chills, weight loss, malaise, arthritis/arthralgia, lymphadenopathy, hepatosplenomegaly, and hearing loss [10]. In the sub‐acute stage, brucellosis shows no significant signs or symptoms and is diagnosed by positive serological tests. The chronic stage occurs when symptoms persist longer than 6 months. In the chronic brucellosis stage, multiple organs may be affected, leading to orchitis, hepatitis, arthritis, endocarditis and encephalomyelitis, and so forth [11]. In particular, the reactivating and chronic nature of *Brucella* infection, along with the pathogen's stealthy intracellular lifecycle, makes this infection difficult to eradicate and requires lengthy antibiotic therapy [8]. It is thus extremely important to understand the host immune response during disease to better design appropriate therapeutic interventions for brucellosis patients. However, a detailed investigation into the immune response landscape in human brucellosis is still lacking.

scRNA‐seq is a powerful technique used to dissect the host immune response [12], and has been used for various infectious diseases (e.g., COVID‐19 [13], tuberculosis [14]), but has not yet been used for brucellosis. Here, we present the scRNA‐seq analysis for a cohort of 35 participants, including brucellosis patients in the acute phase (AC: *n* = 10), sub‐acute phase (SA: *n* = 9) and chronic phase (CH: *n* = 10), as well as healthy donors (HD: *n* = 6). We describe the high‐resolution transcriptomic changes in peripheral blood immune cells at different disease phases and highlight the relationship between the disease phase and the host immune response. We also discover important changes to the clinical hallmarks of brucellosis and provide a significant resource to dissect the inflammatory features in brucellosis patients. Together, our data and findings may facilitate a better understanding of the pathogenic and protective immune responses of brucellosis and have important implications for controlling this disease.

## RESULTS

### Integrated analysis of brucellosis scRNA‐seq data

**Figure 1:**
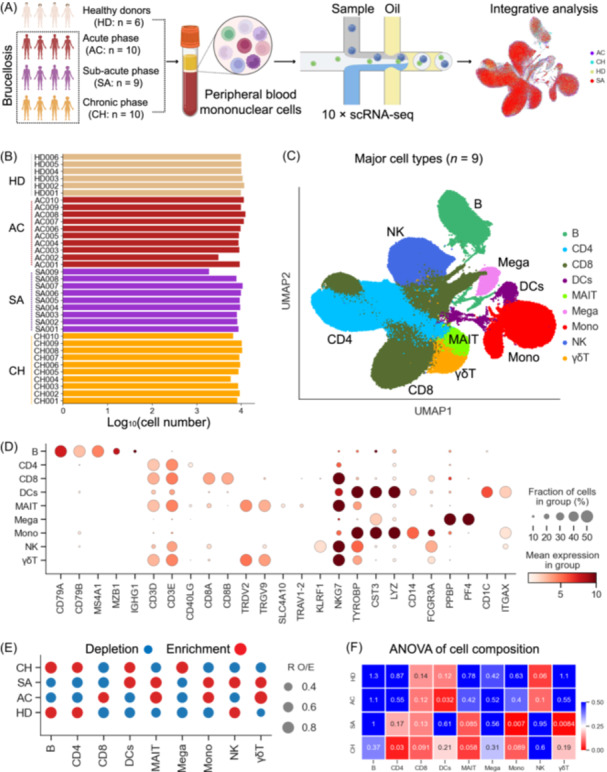
An overview of the results and study design for our peripheral blood mononuclear cell (PBMC) single‐cell transcriptomic study. (A) Diagram outlining the overall study design. Thirty‐five samples were obtained from 35 individuals, including 29 brucellosis patients (10 patients in acute phase, nine patients in sub‐acute phase, and 10 patients in chronic phase) and six healthy donors. (B) Box plots illustrating the log_10_ transformed number of cells for each sample. (C) The clustering result (left row) of the nine major cell types (right row) from 35 samples. Each point represents one single cell, colored according to cell type. (D) Dot plots of the nine major cell types (columns) and expression of their marker genes (rows). (E) Disease preference of major cell clusters as estimated using RO/E. (F) Heatmap showing the association between cell composition and disease types. The color represents analysis of variance (ANOVA) *q* values.

To gain insights into the host immune response to brucellosis, we conducted scRNA‐seq to investigate the transcriptomic profiles of peripheral blood mononuclear cells (PBMCs) obtained from 29 patients and six healthy control donors (HDs) (Figure 1A). The 29 patients with brucellosis were classified into three clinical stages: acute stage (*n* = 10), sub‐acute stage (*n* = 9) and chronic stage (*n* = 10). The laboratory findings and clinical features of enrolled brucellosis patients are provided in Table S1. Strict quality controls were used to ensure that the data generated were from single and live cells (Figure S1A–C, see Methods). Due to inadequate median gene counts, unique molecular identifiers (UMIs) and cell numbers, two PBMC samples (AC002 and SA009) did not pass quality control (Figure 1B). Hence, the analysis contained 33 samples, including 27 brucellosis patients and six healthy controls. After filtering the scRNA‐seq data, a total of 290,369 cell transcriptomes, including 84,221 cells from the AC condition, 67,296 cells from the SA condition, 80,589 cells from the CH condition and 58,263 cells from the HDs, were retained for subsequent analysis across the 33 participants (Figure 1B). Each PBMC sample generated around 8799 cells on average (Figure 1B).

Unsupervised clustering using uniform manifold approximation and projection (UMAP) and canonical marker gene expression identified nine major cell lineages (Figure 1C,D and Table S2): B cells, CD4^+^ T cells, CD8^+^ T cells, mucosal‐associated invariant T cells (MAIT), γδ T cells (γδ T), natural killer cells (NK), dendritic cells (DCs), monocytes and megakaryocytes. The major cell lineages (*n* = 9) identified here encompassed diverse cell clusters in the peripheral blood (Figure 1C, Figure S1D), and notable differences could be observed according to UMAP (Figure 1A). Using *R*_O/E_ analysis [14], the disease preference of nine major cell lineages was assessed (Figure 1E, Figure S1E–I). The abundance of B and CD4^+^ T cells decreased in acute and sub‐acute patients but were restored in chronic patients (Figure 1E, Figure S1E–I). This is consistent with previous findings that the levels of CD3^+^CD4^+^ T lymphocytes in brucellosis patients were significantly reduced in comparison to healthy control [15]. In contrast, innate immune cells, including monocytes, MAIT and γδ T cells, were enriched in acute and sub‐acute patients, and reduced in chronic patients (Figure 1E, Figure S1E–I). The increase in monocytes in PBMCs from brucellosis patients has been observed in a previous study [16], and this supports the accuracy of our scRNA‐seq analysis. The preference of megakaryocytes, CD8^+^ T and NK cells in distinct disease conditions was also depicted, with megakaryocytes, CD8^+^ T cells and NK cells being enriched in chronic, acute and sub‐acute patients, respectively (Figure 1E, Figure S1E–I). In addition, we utilized analysis of variance (ANOVA) to examine the association between disease conditions and compositional changes of the nine major cell lineages (Figure 1F). Multiple immune cell lineages (e.g., CD4^+^ T, DCs, monocytes) were associated with specific brucellosis disease stages, that is, DCs, monocytes and CD4^+^ T cells were significantly associated with acute, sub‐acute and chronic patients, respectively. These findings indicate that each brucellosis disease stage may be linked to a unique immune signature.

### Association of disease phase with various immune cell compositions

**Figure 2:**
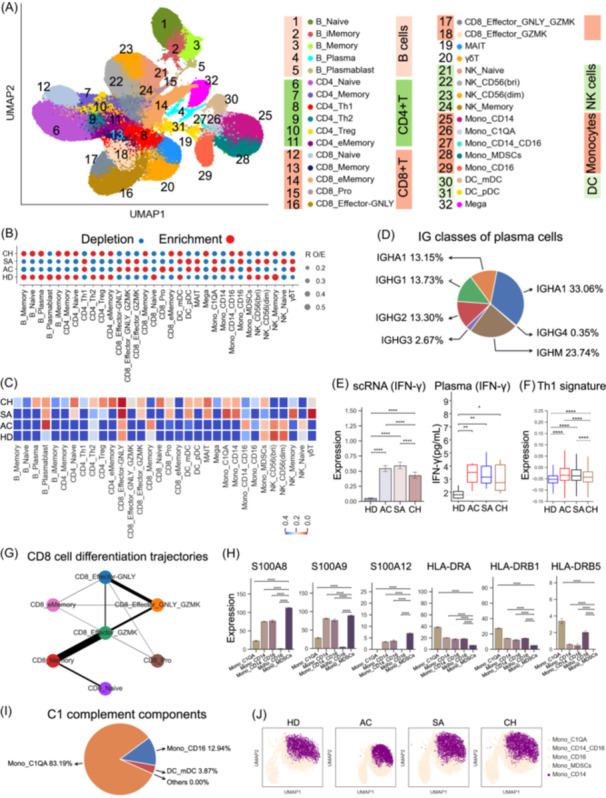
Associations between brucellosis disease phase and peripheral blood mononuclear cell (PBMC) cellular composition. (A) UMAP projection showing the 32 cellular subtypes identified from 35 samples. Each dot depicts a single cell while the color represents the cell subtype. (B) Dot plot depicting the disease preference for each of the 32 cell subtype as calculated using RO/E. (C) Heatmap showing the *p* values from analysis of variance (ANOVA) of differences in cell subtype composition between disease phases. Disease phase: HD, AC, SA, and CH. (D) Classes of heavy chains for plasma cells from brucellosis patients. (E) Bar plots (left) showing IFNG expression in CD4_Th1 cells between different groups, Box plots (right) showing plasma level of IFN‐γ across different phases. (F) Bar plot showing Th1 signature expression in CD4_Th1 cells between different groups. (G) PAGA analysis of CD8^+^ T cell pseudo‐time: the associated cell type and the corresponding status are listed. (H) Bar plots showing S100A8/A9/A12 and HLA‐DRA/B1/B5 expression in Mono_MDSCs between different groups. (I) Pie chart depicting the relative contribution of each cell subtype to the C1 complement components. (J) UMAP projection density plots of Mono_CD14 cells from different groups.

To dissect the heterogeneity and functional diversity with each cell lineage, we conducted a sub‐clustering analysis to explore the cell subsets within each of the major nine cell lineages (Figure 2A, Figure S2). Thirty‐two subtypes were identified (B cells and monocytes were each divided into five subtypes, CD4^+^ T and CD8^+^ T cells were each divided into six cell subsets, NK cells were divided into four subsets and DC cells were divided into two subsets), which covered various immune cell clusters in the peripheral blood (Figure 2A, Figure S2). As such, our results provide a delineation of the immune landscape in brucellosis, enabling correct annotation and analysis of these cell subtypes at distinct resolutions.

This information‐rich data set enabled us to highlight the impact of different disease phases on the composition of cell subsets by employing R_O/E_ (Figure 2B,C). Significant associations were detected after multiple testing corrections (Figure 2C). Notably, the majority of B cell subsets displayed associations with chronic brucellosis patients, especially for plasma cells (B_Plasma) and plasmablast cells (B_Plasmablast) (Figure 2C). B_Plasma exhibited high expression of *CD38*, *XBP1*, *IRF4*, and *PRDM1*, confirming their identity as plasma cells (Table S2). Plasma B cells were found to be more abundant in brucellosis patients during the chronic phase (Figure 2B). B_Plasma cells showed high expression of genes encoding the constant regions of immunoglobulin G1 (IgG1), IgG2, IgA1, IgA2 and IgM (Figure 2D), suggesting their function in the production of antigen‐specific antibodies. The B_Plasma cells in PBMCs seemed to arise from active proliferation B cells (B_Plasmablast) according to PAGE (Partition‐based graph abstraction) analysis (Figure S3B). Plasmablast B cells (B_Plasmablast) displayed high *MKI67* and *TMYS* expression which is indicative of their proliferative state (Table S2), were found to be decreased in all brucellosis patients (Figure 2B). The memory B cells (B_Memory), which were the unique source of B_Plasmablast, appeared to be derived from B_iMemroy (an intermediate transition memory B subtype) (Figure S3B). Interestingly, we also identified an association and enrichment of memory B cells (B_Memory and B_iMemory) in chronic brucellosis patients (Figure 2B,C). These findings shed light on the association between B cell clusters and disease phases.

Among CD4^+^ T‐cell subsets, Th1 cells have a fundamental role in conferring an effective immune response against brucellosis [17], and increased frequencies of this subset (CD4_Th1) were observed in acute and sub‐acute brucellosis patients (Figure 2B), consistent with a previous report [17]. Effective elimination of *Brucella* infection relies on the secretion of IFN‐γ cytokine by Th1 cells. Brucellosis patients show significantly elevated levels of IFN‐γ expression relative to healthy donors, and higher IFN‐γ expression is found in acute and sub‐acute brucellosis patients (Figure 2E). These findings were in accordance with previous observations that a lower level of IFN‐γ was detected in patients with chronic brucellosis in comparison to those with acute brucellosis [18]. Procarta cytokine results obtained from plasma further supported the observation that chronic patients had lower levels of IFN‐γ (Figure 2E). In line with IFN‐γ expression, we also detected significantly upregulated expression of Th1 signatures in brucellosis patients, particularly in those from the acute and sub‐acute phases (Figure 2F). Similar patterns of IFN‐γ expression were also seen in CD8^+^ T cells and NK cells, which are additional sources of IFN‐γ (Figure S3C). These findings imply that, compared to acute and sub‐acute brucellosis patients, the attenuated levels of IFN‐γ and Th1 signature might be related to the inadequate immune response to *Brucella* infection in chronic brucellosis patients. Further investigation indicated that Th1 cells (CD4_Th1) were mostly derived from CD4^+^ T effector memory cells (Figure S3D), which were enriched in chronic brucellosis patients (Figure 2B). The reduction in Th1 cells and increase in their precursor further suggests an aberrant Th1 response in chronic brucellosis patients.

For CD8^+^ T cells, most cell subtypes were more enriched in acute patients, and exhibited a decline in sub‐acute and chronic patients (Figure 2B). These CD8^+^ T‐cell subsets displayed distinct associations with disease phases (Figure 2C). A proliferative CD8^+^ T‐cell subset (CD8_Pro), characterized by high expression of *MKI67* and *TYMS*, was obviously increased in acute brucellosis patients (Figure 2B). Using PAGE analysis, the CD8_Pro subset was characterized as a separate branch and appears to be derived from two effector cell subtypes (CD8_Effector_GZMK and CD8_Effector_GNLY) (Figure 2G), with CD8_Effector_GZMK serving as the primary contributor to the CD8_Pro cell pool. The CD8_Effector_GZMK subset was identified as a transitional state, connecting to all other clusters with naïve and initial memory to activated CD8^+^ T‐cell subsets (Figure 2G). The enrichment of CD8_Pro cells and their precursor cells (CD8_Effector_GZMK) in acute patients might be indicative of an intense CD8^+^ T response. In contrast, the decline in the CD8_Pro subset and its precursor in chronic patients may partially indicate a subdued CD8^+^ T response. The trend found in the CD8_Pro subset was mirrored by innate immune T cells, including MAIT and γδ T cells, which showed an enrichment in acute patients and a reduction in those with chronic brucellosis (Figure 2B), implying a unified cytotoxic T‐cell response in *Brucella* infection.

Further clustering of myeloid cells yielded 8 subsets, and most monocyte clusters were enriched in acute patients (Figure 2B). Further investigation revealed that myeloid cell subsets showed distinct associations with each disease stage (Figure 2C), that is, DC subsets showed notable association with chronic patients while the intermediate monocytes (Mono_CD14_CD16) exhibited an association with acute patients. Among myeloid cell subtypes, we identified a myeloid‐derived suppressor cell (MDSC) subtype, which had the phenotype CD14^+^HAL‐DR^‐/lo^ and high expression of calprotectin (e.g., *S100A8/9/12*). MDSCs were derived from clinical monocytes (Mono_CD14), confirming this cluster as monocytic MDSCs (Figure 2H, Figures S2 and S3E). MDSCs, as a heterogeneous group of immature myeloid cells, are increased during inflammation and have the capacity to suppress T‐cell responses [14]. We found that the Mono_MDSCs cluster was more enriched in acute patients (Figure 2B, Figure S3G), implying that monocytes in acute patients highly resembled MDSCs. This finding suggests that MDSCs (Mono_MDSCs) may potentially contribute to immune paralysis in acute patients. Apart from monocytic MDSCs, we identified another monocyte subset (Mono_C1QA), which had high expression of *C1QA/B/C* (Figure 2A, Figure S2), and were increased in acute and sub‐acute patients. Further investigation confirmed that this subset was the primary peripheral contributor to C1 complements (Figure 2I). In acute brucellosis patients, the expression of genes encoding C1 complement components (*C1QA/B/C*) was significantly elevated when compared to those in sub‐acute and chronic stages as well as healthy individuals (Figure S3I). This indicates the potential diagnostic value of these components in acute cases. Clinical monocytes (Mono_CD14) are the predominant subtype of monocytes (Figure S3I), distinct UMAP projection patterns of this cluster between brucellosis patients and healthy controls suggest perturbed transcriptome features (Figure 2J), especially for those in the acute stage.

### Monocyte subtypes are crucial peripheral sources of potential cytokine storms in acute brucellosis

**Figure 3:**
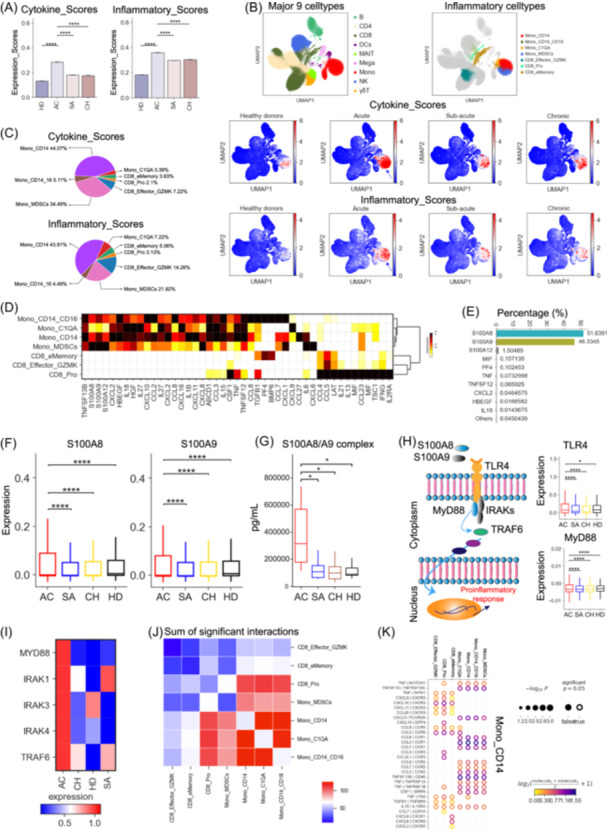
Contribution of *S100A8/A9* to potential cytokine storms in acute patients. (A) Bar plots showing cytokine scores and inflammatory scores across different groups. (B) Uniform manifold approximation and projection (UMAP) projections of peripheral blood mononuclear cells (PBMCs). Colored based on the nine major cell types (top left), seven hyper‐inflammatory cell subtypes (top right), cytokine (middle) and inflammatory score (bottom). (C) Pie charts depicting the relative contribution of each inflammatory cell subtype to the cytokine and inflammatory scores. (D) Heatmap depicting the expression of cytokines within each hyper‐inflammatory cell subtype identified. (E) Bar chart depicting the relative contribution of the top 10 cytokines in patients with acute brucellosis. (F) Box plots showing *S100A8* and *S100A9* expression across different groups. (G) Box plots showing plasma profiling of S100A8/A9 complex across different groups. (H) The expression analysis of S100A8/A9‐TLR4‐MyD88 pathway. (I) Heatmap depicting the expression of selected genes across different groups. (J) Heatmap of the sum of significant interaction among the seven hyper‐inflammatory cell subtypes. (K) Circos plot depicting the ligand‐receptor pair interactions between Mono_CD14 and the seven hyper‐inflammatory cell subtypes.

As inflammation is a typical hallmark of brucellosis [19], we attempted to explore the possible origins of cytokine production in brucellosis. Based on the expression of cytokine and inflammatory genes (Table S3) [14], we assigned a cytokine score and inflammation score to each cell subset, respectively (Figure S4), and utilized these two interconnected scores as a metric to gauge the potential contribution of each cell to the inflammatory response in brucellosis. We observed significantly increased expression of cytokine and inflammatory genes in brucellosis patients compared to healthy donors (Figure 3A,B), confirming that *Brucella* infection triggers a pro‐inflammatory response. In particular, the expression of cytokine and inflammatory genes in sub‐acute and chronic patients did not decrease to levels observed in healthy donors (Figure 3A,B), implying that these patients may suffer from long‐lasting inflammation. Brucellosis patients at the acute stage had the highest expression of cytokine and inflammatory genes, which were significantly higher than those at the sub‐acute and chronic stages as well as healthy controls (Figure 3A,B). This indicates the likelihood of a high‐grade inflammation or a potential inflammatory cytokine storm in these patients.

According to our scRNA‐seq data, fourteen cell subsets, including four monocytes, six T cells and four NK cells, exhibited significantly elevated cytokine and inflammatory score (Figure S4A), suggesting that these cell subsets were in a high inflammatory state. Among these highly inflammatory cell clusters, seven cell clusters, including three CD8^+^ T clusters (CD8_Pro, CD8_Effector_GZMK and CD8_eMemory) and four monocyte clusters (Mono_CD14, Mono_C1QA, Mono_CD14_CD16 and Mono_MDSCs), were detected with significantly higher cytokine and inflammatory sores in acute patients than those at the sub‐acute and chronic stage as well as healthy donors (Figure 3B, Figure S4B). This indicates that these cell clusters may be principal sources of potential inflammatory storms in acute brucellosis. We then examined the relative abundance of each of the seven cell subsets in brucellosis patients, and observed that these inflammatory cell subsets were significantly elevated in patients from the acute phase (Figure 2B, Figure S5A).

Our further analysis confirmed that Mono_CD14 (classical monocytes) and Mono_MDSCs were the major contributors to the potential inflammatory storm present in acute brucellosis patients (Figure 3C). In line with this, CD14‐expressing monocytes (e.g., Mono_CD14 and Mono_MDSCs) have been substantiated as major sources of inflammatory storm in other infectious diseases (e.g., tuberculosis and COVID‐19) [14]. We next analyzed the inflammatory signatures for each identified inflammatory cell cluster and observed unique pro‐inflammatory cytokine gene expression in each inflammatory cell cluster (Figure 3D), such as *S100A8/912*, *TNF*, *CSF1*, *CCL5*, *CXCL8*, *IL6*, *CCL2*, and *CCL8*. Additionally, we also detected high expression of typical inflammatory cytokines (e.g., *S100A8/9/12*, *IL1B*, *IL6*, *CXCL8*, *CCL2*, *CXCL10*) in brucellosis patients during the acute phase (Figure S5B). These findings imply that the potential inflammatory storm in acute patients might be driven by different mechanisms. Two inflammatory cell subsets, including Mono_CD14 and Mono_MDSCs, largely expressed more cell‐type‐specific pro‐inflammatory cytokines (Figure 3D), further verifying their central role in driving the potential inflammatory storm present in acute patients.

Ten pro‐inflammatory cytokines, including *S100A8/9/12*, *MIF*, *PF4*, *TNF*, *TNFSF12*, *CXCL2*, *HBEGF* and *IL18*, may be the major contributors of potential inflammatory storm, because these cytokines contributed to >99% of the cytokine scores in acute patients (Figure 3E). Interestingly, further investigation found that these top 10 most highly expressed pro‐inflammatory cytokines were mainly expressed in Mono_CD14 and Mono_MDSCs (Figure S5C). Among these top 10 pro‐inflammatory cytokines, S100A8/A9, mainly secreted by Mono_CD14 and Mono_MDSCs (Figure S5D), might play a central role in driving the inflammatory storm as they contributed ~98% to the cytokine scores (Figure 3E). Notably, brucellosis patients at the acute stage showed a significant increase in the expression of *S100A8/A9* genes (Figure 3F), providing further evidence for our hypothesis. For this cohort, we also measured the cytokine levels in plasma, which supports our observation that acute patients had higher levels of S100A8/A9 complex (Figure 3G). This further confirmed the precision and reliability of our scRNA‐seq analysis. These data highlight the importance of the hyper‐inflammatory Mono_CD14 and Mono_MDSCs clusters as well as S100A8/A9 for developing potential therapeutic interventions to ameliorate the immunopathogenesis in acute *Brucella* patients.

S100A8/A9 molecules (also known as MRP8/P14), mainly released by monocytes, neutrophils and macrophages during infection, can modulate inflammation by inducing pro‐inflammatory cytokines [20]. Consistently, monocytes, especially for Mono_CD14 and Mono_MDSCs, were the major sources of S100A8/A9 proteins in peripheral blood in acute brucellosis patients (Figure S5D). S100A8/A9 molecules bind to toll‐like receptor 4 (TLR4) and trigger the MyD88‐dependent signaling pathway, which is crucial for inflammation (e.g., inducing the release of multiple cytokines in inflammatory cells) [20]. The expression of *TLR4* was significantly elevated, especially in inflammatory Mono_CD14 and Mono_MDSCs cells, in acute patients compared to those from healthy controls, sub‐acute and chronic stages (Figure 3H). *S100A8/A9*‐*TLR4* signaling initiates the MyD88‐dependent pathway by inducing translocation of *MyD88*, hyperphosphorylation of *IRAKs*, and activation of *TRAF6*, resulting in the augmentation of pro‐inflammatory response and extensive tissue damage (Figure 3H). We found that key genes in the MyD88‐dependent signaling pathway were notably increased in acute patients (Figure 3I), particularly in inflammatory monocytes (Figure S5F). These results indicate that brucellosis patients at the acute stage exhibited S100A8/A9‐TLR4‐inflammatory traits, highlighting the significance of S100A8/A9 for developing effective therapeutic approaches to mitigate immunopathogenesis in acute brucellosis patients.

The potential inflammatory storm in brucellosis may be linked to cellular cross‐talk between hyperinflammatory cell subsets through the release of a wide array of cytokines [14]. We thus investigated the ligand‐receptor pairing patterns of seven hyperinflammatory cell subsets from acute brucellosis patients (Figure 3J,K, Figure S5G,H). Several notable ligand‐receptor interactions were detected within the seven hyperinflammatory subsets (Figure 3J). CD14‐expressing monocytes exhibited more interactions with each other than CD8‐expressing cells (Figure 3I). Two core inflammatory cell subsets, including Mono_CD14 and Mono_MDSCs, expressed multiple receptors (e.g., *CCR1*, *CCR2*, *CCR3*, *CCR5*, *CXCR3*, *CCR10*, *CXCR1*, *CXCR2*), suggesting that these two cell subsets have the ability to respond to multiple cytokines secreted from other cells (Figure 3I,J, Figure S5G,H). Interestingly, our data confirmed that the interactions between Mono_CD14/Mono_MDSCs and other inflammatory cell clusters may primarily be dependent on chemokines and their receptors (Figure 3I,J, Figure S5G,H). Collectively, these results shed light on the potential molecular mechanisms underlying the interactions among hyperinflammatory cell subsets in acute patients.

### The dysregulated Th1 response in chronic brucellosis patients

**Figure 4:**
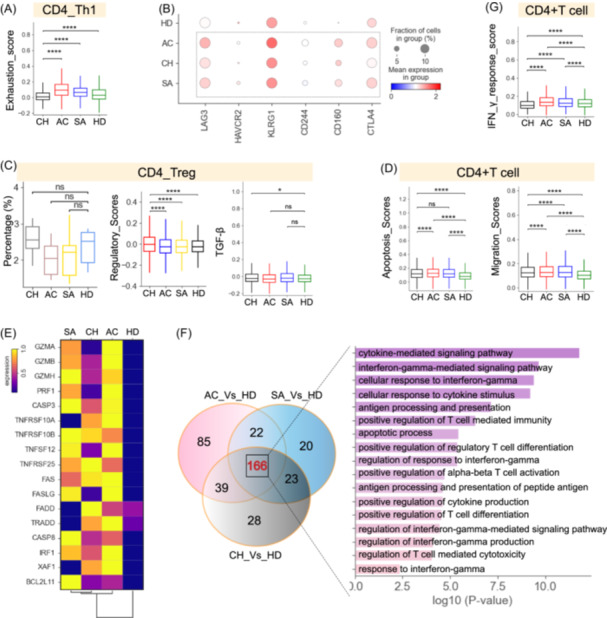
Immunological features of CD4^+^ T‐cell subsets. (A) Box plot showing the exhaustion score in CD4_Th1 cell subtype across different groups. (B) Dot plot showing the expression of selected exhaustion genes in CD4_Th1 cell subtype across different groups. (C) Box plot showing the relative percentage (left), regulatory scores (middle) and TGF‐β expression of CD4_Treg cell subtype across different groups. (D) Box plot showing the apoptosis and migration scores of CD4^+^ T cells from different groups. (E) Heatmap depicting the expression of apoptosis‐related genes across different groups. (F) Venn diagram (left) illustrating the number of upregulated genes in CD4^+^ T cells and box plots (right) of shared GO terms of CD4^+^ T cells across different conditions. (G) Box plot showing the indicated functional scores (IFN‐γ response scores) of CD4^+^ T cells.

A total of six CD4^+^ T‐cell subtypes were identified (Figure 2, Figure S2). Two different fates for CD4^+^ T cells were confirmed by PAGA trajectory analysis, with CD4_Th1 and CD4_Memory as different ends (Figure S3D). The developmental trajectory was found to be correlated with the functional status in diverse cell subtypes (Figure S6A). By investigating the signature genes reported previously [14], we found different functional statuses for each CD4^+^ T‐cell subtype, with the highest naïve score for the CD4_Naive subset. The highest inflammatory scores were found in Th1, Treg, and effector memory subsets, while the highest cytotoxicity and exhaustion scores were seen in CD4_Th1 subtypes (Figure S6A). Further analysis found that Th1 cells had the highest exhaustion scores in brucellosis patients (Figure 4A), and highly expressed multiple inhibitory receptors (e.g., *LAG3*, *CD160*, *CTLA4*) (Figure 4B). These results indicate Th1 cell exhaustion in brucellosis, especially for those in the chronic stage. This might be associated with inefficient control of persistent *Brucella* infection.

Disease preference analysis revealed that CD4_Th1 increased in the acute and sub‐acute stages, while the rest of the five CD4 clusters, including Treg cells (CD4_Treg), were mainly found in the chronic stage (Figures 2B and 4C). In particular, analysis of the regulatory effector score in CD4_Treg cells across different stages revealed high levels of regulatory effector function in chronic patients (Figure 4C, Figure S6B). Treg cells produce TGF‐β, leading to the inhibition of CD4^+^ T cell responses, the suppression of T‐cell cytokine generation, and the downregulation of effector‐immune responses [21]. Interestingly, Treg cells (CD4_Treg) highly expressed TGF‐β in chronic patients (Figure 4C). These results indicated that immune regulation by Treg cells (CD4_Treg) may also be related to immune tolerance and *Brucella* persistence in chronic patients.

Utilizing an apoptosis scoring system [14], we found that CD4^+^ T cells in brucellosis patients likely underwent apoptosis relative to healthy donors (Figure 4D), consistent with previous findings that *Brucella* can induce apoptosis of human T lymphocytes [22]. Four CD4^+^ T‐cell subsets, including CD4_eMemory, CD4_Treg, CD4_Th2 and CD4_Th1, may likely have undergone apoptosis (Figure S6C). Genes involved in granzyme/perforin, FAS, TNF, and XAF1 apoptosis pathways were significantly upregulated (e.g., *GZMB*, *CASP3*, *FAS*, *XAF1*, *TNFSF12*) (Figure 4E), indicating that the apoptosis of CD4^+^ T cells may be caused by granzyme/perforin, FAS, TNF and XAF1 apoptosis pathways. Similar to our findings in apoptosis, significant activation of cell migration pathways in CD4^+^ T cells was also observed in brucellosis patients, with high migration scores in CD4_eMemory, CD4_Treg, CD4_Th1, CD4_Th2 and CD4_Memory (Figure 4D, Figure S6C).

We then performed the transcriptome analysis of CD4^+^ T cells in brucellosis patients. Compared to healthy individuals, we found 334, 345, and 346 upregulated differentially expressed genes (DEGs) in acute, sub‐acute, and chronic patients, respectively, of which 166 DEGs were common (Figure 4F, and Table S4). Gene Ontology (GO) analyses revealed that the commonly upregulated genes were involved in ‘interferon‐gamma response’ (e.g., ‘response to interferon‐gamma’ and ‘cellular response to interferon‐gamma’) (Figure 4F). This is in agreement with the concept that IFN‐γ responses are crucial for the immune response to *Brucella* infection. Consistently, these genes associated with ‘interferon‐gamma response’ (e.g., *IFNG*, *IRF1*, *SOCS1*) are also enriched in brucellosis patients (Figure S6D). However, analysis of the IFN‐γ response score in CD4^+^ T cells suggest higher levels of IFN‐γ response function in acute and sub‐acute patients relative to chronic patients (Figure 4G), implying that a reduced IFN‐γ response might also be associated with *Brucella* persistence in chronic patients.

### The dysregulated CD8 response in chronic brucellosis patients

MAIT and γδ T cells had low expression of CD8A gene (Figure 1D), which categorized them as CD8^+^ T cells for transcriptomic analysis. The CD8^+^ T cells were further clustered into nine subsets (Figure 2A), including naïve (CD8_Naive), effector (CD8_Effector_GNLY, CD8_Effector_GNLY_GZMK and CD8_Effector_GZMK), effector memory (CD8_eMemory), memory (CD8_Memory), MAIT, γδ T and a proliferating subtype (CD8_Pro). Each CD8^+^ T subtype displayed different disease preferences: the naïve cluster was enriched in healthy donors, while the rest were mainly found in acute and sub‐acute patients (Figure 2B). These data indicate that the reduced frequencies of cytotoxic T cells in chronic patients might be linked to *Brucella* persistence.

**Figure 5:**
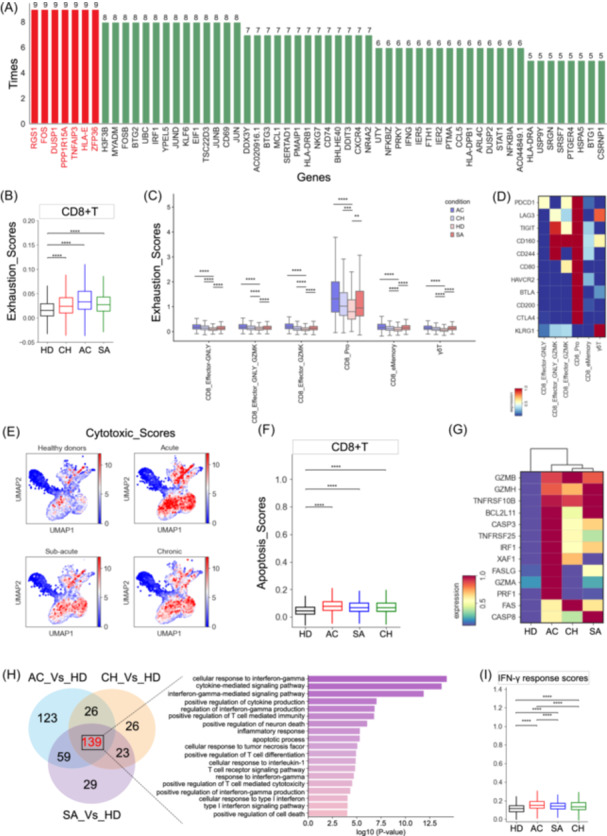
Immunological features of CD8^+^ T‐cell subsets. (A) Top upregulated genes for each CD8^+^ T‐cell cluster was calculated, and genes with high frequencies are displayed. (B) Box plot showing the exhaustion scores in CD8^+^ T cells across different groups. (C) Box plots showing the exhaustion score in effector CD8^+^ T cells from different groups. (D) Heatmap depicting the expression of exhaustion‐related genes in effector CD8^+^ T cells. (E) Uniform manifold approximation and projection (UMAP) projections for cytotoxic scores in CD8^+^ T cells across different conditions. (F) Box plot showing the apoptosis scores in CD8^+^ T cells across different groups. (G) Heatmap depicting the expression of apoptosis‐related genes in CD8^+^ T cells across different groups. (H) Venn diagram (left) illustrating the number of upregulated genes in CD8^+^ T cells and box plots (right) of shared GO terms of CD8^+^ T cells across different conditions. (I) Box plot showing the IFN‐γ response scores of CD8^+^ T cells from different cells.

We next compared the transcriptional characteristics of each cluster between patients and healthy donors. Interestingly, a series of commonly upregulated genes were identified in brucellosis patients, with seven genes/transcription factors (*RGS1*, *DUSP1*, *FOS*, *PPP1R15A*, *TNFAIP3*, *HLA‐E*, and *ZFP36*) being the most frequent (nine times among nine clusters) (Figure 5A, Figure 7SA). It has been reported that these commonly upregulated genes are primarily involved in T‐cell exhaustion (e.g., *RGS1*) [23], immunosuppression (e.g., *DUSP1* [24], *PPP1R15A* [25]) and cell apoptosis (e.g., *FOS* [26], *TNFAIP3* [27]), indicating the diverse mechanisms which may potentially lead to *Brucella* persistence. Further studies are needed to clarify the significance of other commonly upregulated genes.

Upregulated RGS1 (Figure 5A, Figure S7A), which mediates T‐cell retention, results in persistent antigen stimulation [28] and leads to T‐cell exhaustion [23]. It is well known that exhausted T cells are involved in negative regulation of the immune system, causing immune escape. Here, we next determined the exhaustion status of CD8^+^ T‐cell subclasses in brucellosis patients, and found that CD8^+^ T cells had a significantly higher exhaustion state in brucellosis patients compared to healthy individuals (Figure 5B). Six subtypes, including CD8_Pro, CD8_eMemory, CD8_Effector_GZMK, CD8_Effector_GNLY, CD8_Effector_GNLY_GZMK and γδ T, exhibited higher exhaustion scores compared to the other clusters (Figure S7B). This implies that these subtypes may be the main exhausted CD8^+^ T cells. Within these exhausted subclasses, the lowest exhaustion scores were observed in healthy individuals, while patients with brucellosis displayed a higher level of exhaustion (Figure 5C). We then examined the exhaustion signatures for each exhausted cell subset and found each exhausted CD8^+^ T‐cell subset had different inhibitor molecules expressed (Figure 5D), such as *PD‐1*, *LAG3*, *HAVCR2*, and *BTLA*. This suggests that there may be multiple mechanisms which lead to CD8^+^ T‐cell exhaustion in *Brucella*‐infected patients.

Cytotoxic T cells play a vital role in controlling intracellular infections by releasing effector molecules (e.g., granzyme). We thus examined the cytotoxic status of CD8^+^ T cells and found that the highest cytotoxic scores were in acute patients. In contrast, chronic patients had relatively low cytotoxic scores (Figure 5E), which may partially affect the ability of cytotoxic T cells to control brucellosis in these patients. Four subclasses, including three effector CD8^+^ T‐cell subsets (CD8_eMemory, CD8_Effector_GNLY and CD8_Effector_GNLY_GZMK) and γδ T, may be the major contributors for eliminating the infected host cells in brucellosis patients (Figure S7B) as they simultaneously expressed multiple effector molecules (e.g., *GZMA*, *GZMK*, *GNLY*, *CST7*), especially for those at the acute stage (Figure S7C). Interestingly, these CD8^+^ T cells, which exhibit high cytotoxicity, are also characterized as exhausted cells (Figure 5). This finding is consistent with earlier functional studies on exhausted CD8^+^ T cells, which have validated that, in contrast to their reduced proliferative capacity and cytokine production, their cytotoxic states remain unaffected [29].

In addition to their roles in directly killing the infected targets, cytotoxic T cells also induce cell apoptosis, which is granzyme/perforin or Fas‐Fas ligand‐mediated. The apoptosis scoring system revealed that CD8^+^ T cells in brucellosis patients displayed a higher apoptosis score relative to healthy donors (Figure 5F), suggesting that *Brucella* infection can induce apoptosis of human CD8^+^ T lymphocytes, in agreement with the above transcriptional profiles (Figure 5A). CD8^+^ T cells in acute patients had the highest apoptosis scores, being more prone to apoptosis (Figure 5F). The apoptosis trend was further evident in seven CD8^+^ T‐cell subtypes (e.g., CD8_Pro, MAIT, CD8_Effector_GNLY) (Figure S7D). We further analyzed the expression of genes in apoptosis‐related granzyme/perforin, TNF, XAF1, and FAS pathway [14], and found that, in CD8^+^ T cells, most granzyme/perforin, TNF, *XAF1* and *FAS* pathway members (e.g., *GZMB/H*, *XAF1*, *TNFRSF10B*, *IRF1*, *FAS*, *CASP3/8*) exhibited an upregulated trend in brucellosis patients (Figure 5G), especially for those from the acute stage. The upregulated genes in granzyme/perforin, TNF, XAF1 and FAS apoptosis pathways may result in elevated apoptosis of CD8^+^ T cells in brucellosis patients and thus directly inhibit CD8^+^ T‐cell‐mediated responses.

To further investigate the changes of CD8^+^ T cells among brucellosis patients, we conducted a DEG and GO analysis. Our analysis revealed that, in comparison to healthy donors, acute, sub‐acute, and chronic patients had 347, 250, and 214 DEGs upregulated (Figure 5H, respectively Table S5), with a common set of 139 DEGs. The results of GO analyses confirmed that these upregulated genes were involved with ‘cell apoptotic process’ and ‘T‐cell‐mediated cytotoxicity’ (Figure 5H), in accordance with the aforementioned analysis (Figure 5E–G). Interestingly, similar to our findings in CD4^+^ T cells, the common upregulated genes in CD8^+^ T cells also displayed notable enrichment in the ‘interferon response’ pathway, particularly in relation to ‘interferon‐gamma response’ (Figure 5H). In line with this, the enrichment of genes linked to the ‘interferon‐gamma response’ pathway (e.g., *STAT1*, *IFNG*, *IRF1*, *B2M*, *GAPDH*) was also consistently observed in brucellosis patients (Figure S7E). Despite this, acute and sub‐acute patients showed higher levels of IFN‐γ response function relative to chronic patients (Figure 5I), suggesting that the diminished IFN‐γ response in CD8^+^ T cells might also contribute to the persistence of *Brucella* in chronic patients.

### The exhausted NK cells in brucellosis patients

**Figure 6:**
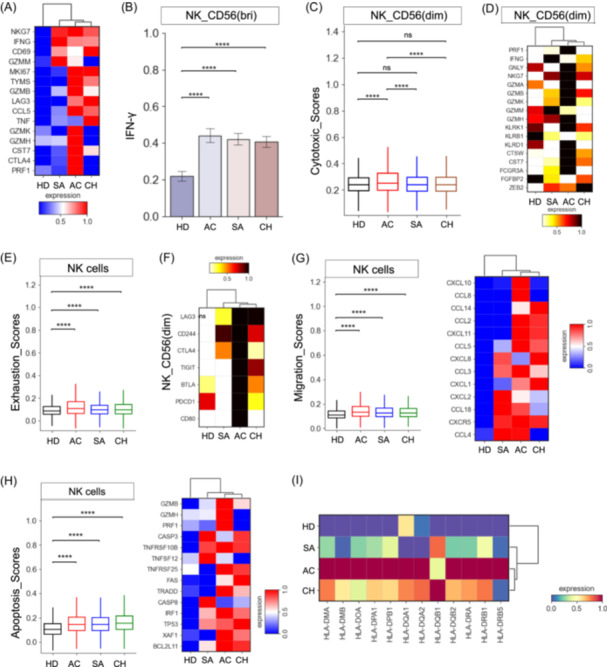
Immunological features of NK‐cell subsets. (A) Heatmap depicting the expression of activation‐related genes in effector NK cells across different conditions. (B) Bar plots showing the IFN‐γ expression in NK_CD56^(Bri)^ cells between different groups. (C) Box plot showing the cytotoxic scores in NK_CD56^(Dim)^ cells across different groups. (D) Heatmap depicting the expression of cytotoxicity‐related genes in NK_CD56^(Dim)^ cells across different conditions. (E) Box plot showing the exhaustion score in NK cells across different groups. (F) Heatmap depicting the expression of exhaustion‐related genes in NK_CD56^(Dim)^ cells across different conditions. (G) Box plot (left) showing the migration score in NK cells across different groups, heatmap (right) depicting the expression of migration‐related genes in NK cells across different conditions. (H) Box plot (left) showing the apoptosis score in NK cells across different groups, heatmap (right) depicting the expression of apoptosis‐related genes in NK cells across different conditions. (I) Heatmap depicting the expression of selected genes in NK cells across different conditions.

Further clustering of NK cells resulted in the subdivision into four distinct subtypes, including naïve (NK_Naive), memory (NK_Memory) and effector NK (NK_CD56^(bri)^ and NK_CD56^(dim)^) (Figure 2, Figure S2). Multiple activation markers, such as *CD69*, *MKI67*, *CCL5*, *CTLA4*, *IFNG* and *GZMB*, were found to be enriched in the NK cells of brucellosis patients, indicating the presence of an activated NK cell response as a distinctive feature for brucellosis patients, especially for those at the acute stage (Figure 6A, Figure S8A). The CD56^bri^ NK subset (NK_CD56^(bri)^) is a potent source of anti‐*Brucella*‐associated cytokines (e.g., IFN‐γ). Similar to our observations in CD4_Th1 cells, the NK_CD56^(bri)^ cluster exhibited significant elevation of IFN‐γ in brucellosis patients in comparison to healthy individuals (Figure 6B). The CD160 NK subset (NK_CD56^(dim)^), which contributes to host defense against *Brucella* through cell‐mediated cytotoxicity, had a significantly higher cytotoxic state in acute patients compared to other groups (Figure 6C, Figure S8B). Moreover, the NK_CD56^(dim)^ subtype in acute patients showed elevated expression of multiple cytotoxic genes, such as *PRF1*, *GNLY*, *NKG7*, *GZMA*, *CST7*, *CTSW*, *KLRD1* (Figure 6D). The elevated cytotoxic state of the NK_CD56^(dim)^ subset in acute patients could potentially cause immunopathology comparable to that observed in CD8^+^ T cells.

To gain further insights into the transcriptomic changes within the NK cell subsets, we then examined the exhaustion, apoptosis, and migration states of different NK cell subsets in active brucellosis patients. At the bulk level, NK cells from brucellosis patients had a higher exhaustion status relative to healthy donors, with the highest exhaustion level observed in acute patients (Figure 6E). Among these NK subsets, the cytotoxic NK cluster (NK_CD56^(dim)^) was identified as exhausted NK cells with higher exhaustion scores (Figure S8C). The exhausted NK cells (NK_CD56^(dim)^) highly expressed multiple inhibitory molecules (e.g., *LAG3*, *CD244*, *CTLA4*) in brucellosis patients compared to healthy donors (Figure 6F). In particular, the persistence of *Brucella* in chronic patients could also be attributed to the exhausted NK_CD56^(dim)^. We found that NK cells, especially for NK_Naive, NK_Memory and NK_CD56^(dim)^, in brucellosis patients potentially underwent migration (Figure 6G, Figure S8D), with several migration‐related genes highly expressed like *CCL4*, *CXCR5*, *CCL18*, *CXCL2*, and so forth (Figure 6G). Likewise, NK cells in brucellosis patients were prone to apoptosis (Figure 6H), particularly in NK_Naive, NK_Memory and NK_CD56^(dim)^ (Figure S8E). Further analysis found that granzyme/perforin, *TNF*, *XAF1*, and *FAS* apoptosis pathways might contribute to apoptosis of NK cells in patients with brucellosis, potentially exerting a direct inhibitory effect on NK cell‐mediated responses.

Compared to healthy controls, we observed an obvious increase in the expression of genes encoding HLA class II molecules in individuals infected with *Brucella* (Figure 6I). A higher degree of upregulation in HLA class II molecules was observed in acute individuals (Figure S8F). The upregulation of HLA class II molecules is mirrored in differentially elevated gene pathways (e.g., enhanced crosstalk between DCs and NK cells). Similarly, an elevated expression of HLA‐I molecules was observed in brucellosis patients relative to other conditions, including canonical HLA‐I genes HLA‐A/B, and noncanonical HLA‐I gene HLA‐E/F (Figure S8G).

### Dysregulated immune response in myeloid cells from brucellosis patients

**Figure 7:**
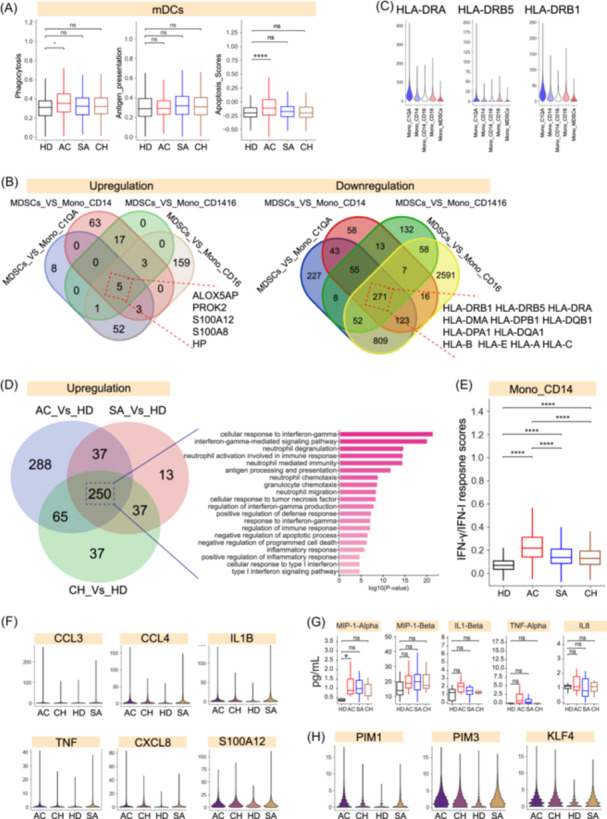
Immunological features of myeloid subsets in brucellosis patients. (A) Box plot showing the phagocytosis (left), antigen presentation (middle) and apoptosis scores (right) in mDCs across different groups. (B) Venn diagram illustrating the number of upregulated genes (left) and downregulated genes in Mono_MDSCs. (C) Violin plots showing the expression of HLA‐DRA/B5/B1 across monocyte subsets. (D) Venn diagram illustrating the number of upregulated genes in classical monocytes (left), selected enriched GO terms (right) for genes upregulated in classical monocytes. (E) Box plot showing the IFN response in clinical monocytes across different groups. (F) Violin plots showing expression of typical inflammatory cytokines in clinical monocytes across different groups. (G) Box plots showing plasma profiling of MIP‐α (CCL3), MIP‐1‐β (CCL4), IL1‐β (IL‐1B), TNF‐α (TNF) and IL‐8 (CXCL8) across different groups. (H) Violin plots showing expression of *PIM1*, *PIM3* and *KLF4* in clinical monocytes across different groups.

Defense against *Brucella* requires the activation of the bactericidal mechanisms in antigen‐presenting cells (APCs) like DCs and macrophages. Classical DCs (mDCs), which specialize in antigen processing, play a critical role in recognizing microbes, initiating innate immune responses and inducing robust adaptive immune responses. In light of this, we investigated the phagocytosis and antigen presentation capacity of mDCs in response to *Brucella* infection. Only acute patients displayed a significantly higher phagocytosis capacity than healthy donors (Figure 7A). Effective antigen presentation relies on the participation of major histocompatibility complex (MHC) class II (HLA‐DR) molecules. No significant upregulation of HLA‐II molecules was observed in brucellosis patients compared to healthy individuals, and a significant downregulation was found in acute patients (Figure S9A). In agreement with this, the expression levels of phagocytosis‐ and antigen‐presentation‐associated genes (e.g., *CIITA*, *RFX5*, *HLA‐DPA1*, *WASF2*) were reduced in brucellosis patients (Figure S9B). Moreover, sub‐acute and chronic patients may not trigger mDCs apoptosis, thus providing protection against immune attacks associated with *Brucella* and allowing the pathogen to multiply optimally within mDCs (Figure 7A). These data indicate that *Brucella* might interfere with phagocytosis, antigen presentation and apoptosis in mDCs to establish chronic infection. In the case of nonprofessional antigen‐presenting cells (APCs) such as B cells and monocytes, we observed a noticeable decline in phagocytosis, antigen presentation, and apoptosis capacity in chronic patients relative to acute patients (Figure S9C,D).

Among myeloid cells, a monocyte cluster (Mono_MDSCs) showed a strong association with acute and chronic patients (Figure 2C, Figure S2). For a more detailed analysis, Mono_MDSCs were characterized by higher expression of inflammatory molecules (e.g., *S100A8*/*A12*) and lower expression of HLA genes, including *HLA‐I* (e.g., *HLA‐A*, *HLA‐B*) and *HLA‐II* (e.g., *HLA‐DRB1*, *HLA‐DPB1*, *HLA‐DMA*) molecules, compared to other monocytes (Figure 7B, Figure S9E). In particular, low expression of HLA‐DR is a recognized surrogate marker of monocyte dysfunction (Figure 7C), leading to a decrease in responsiveness to microbial stimuli [30], suggesting that Mono_MDSCs cluster is the dysfunctional monocytes. Consistent with these findings, the 271 genes that were downregulated in Mono_MDSCs (Figure 7B) displayed notable enrichment in ‘interferon‐gamma response’, ‘antigen processing and presentation’ and ‘negative regulation of T‐cell‐mediated immune’ (Figure S9F), which further supports the characterization of this cluster as dysfunctional monocytes.

Classical monocytes (Mono_CD14), which are the predominant myeloid cell type in PBMCs (Figure S2I), are affected by brucellosis as indicated by UMAP projection patterns of this cluster between brucellosis and controls as well as an enrichment in acute patients (Figures S9G, 2B). Among the DEGs in Mono_CD14, we discovered 640, 337 and 389 upregulated genes in acute, sub‐acute and chronic patients compared to healthy donors (Figure 7D and Table S6). The substantial number of upregulated DEGs indicated that significant differences exist between brucellosis patients and healthy donors. In contrast, we only detected a small number of downregulated DEGs in brucellosis patients relative to controls (Figure S10A). A GO analysis using upregulated DEGs showed that the terms: ‘response to IFN‐γ’, ‘response to IFN‐I’ and ‘inflammation response’ were shared by all brucellosis patients (Figure 7D). Further investigation was conducted on the DEGs linked to these GO terms. In brucellosis patients, the expression levels of many typical IFN‐γ/IFN‐I response genes, including *IFITM3*, *B2M*, *GBP1*, *IRF1*, *IFI30*, *IFNGR2*, and so forth, were higher than those in controls (Figure S10B). However, acute patients exhibited the highest levels of IFN‐γ response function, implying that the reduced IFN‐γ response in classical monocytes may be linked to the prolonged presence of *Brucella* in chronic patients (Figure 7E). Consistent with the previous inflammatory analysis in this study (Figure 3), the GO terms: ‘inflammatory response’ and ‘positive regulation of inflammatory response’, were enriched in all brucellosis patients (Figure 7D). This implies that classical monocytes may contribute to a long‐lasting pro‐inflammatory response in brucellosis and thus mediate tissue damage. As the major contributor of potential inflammatory storm (Figure 3), many inflammatory response genes (e.g., *ITGB2*, *OSM*, *FPR1*, *CEBPB*, *NINJ1*) and canonical pro‐inflammatory cytokines (e.g., *CXCL8*, *CCL3*, *CCL4*, *TNF*, *IL1B*, *S100A12*) were expressed at higher levels in brucellosis patients than in healthy donors (Figure 7F, Figure S10C). Procarta cytokine analysis from the plasma of these individuals (Figure 7G), supported the finding that brucellosis patients, especially for acute patients, had a higher level of multiple pro‐inflammatory cytokines, such as CXCL8/IL8, CCL3/MIP‐α, CCL4/MIP‐β, TNF/TNF‐α, IL1B/IL1‐β (Figure 7G).

Additionally, several GO terms, including ‘negative regulation of apoptotic process’ and ‘negative regulation of programmed cell death’, were also enriched in classical monocytes (Figure 7D), suggesting a potential effect of *Brucella* infection on monocytic apoptosis. The expression levels of multiple genes (e.g., *PIM1*, *PIM3*, *KLF4*) involved in the ‘negative regulation of cell apoptotic process’ were higher in brucellosis patients than in controls (Figure S10D). For instance, PIM1 contributes to cell survival by phosphorylating and inhibiting proapoptotic proteins (Figure 7H) [31]. PIM3 has the ability to prevent cell apoptosis, promote cell survival, and enhance protein translation (Figure 7H) [32]. *KLF4* inhibits cell apoptosis through the p53‐KLF4‐p21‐cyclinD1 axis (Figure 7H) [33]. These findings suggest that *Brucella* infection is able to suppress monocyte apoptosis through multiple mechanisms, potentially resulting in the development of chronic infection. These genes may be potential therapeutic targets for chronic infection.

## DISCUSSION

Brucellosis, is the most common bacterial zoonotic infection globally, impacting more than half a million individuals annually [17]. Nevertheless, a comprehensive global characterization of the anti‐*Brucella* or pathogenic immune responses at different disease phases is still absent. To obtain an unbiased and comprehensive understanding of the immunological characteristics and connections with disease status in *Brucella*‐infected patients, we utilized scRNA‐seq and constructed a comprehensive immune landscape of PBMCs across different *Brucella* stages (AC, SA, CH, and healthy donors (HD)). These results will not only provide valuable insights into the pathogenesis at distinct stages of *Brucella* infection in humans but also aid in the identification of potential immune targets and development of novel therapeutic strategies for the effective treatment of brucellosis, especially for those in the chronic phase.

The major defense mechanism against *Brucella* infection relies on cell‐mediated immunity, which involves the activation of APCs (e.g., mDCs, B cells) and the subsequent amplification of antigen‐specific T‐cell clones, leading to the eradication of this pathogen [34]. Consistent with previous findings, the expansion in CD8^+^ T cells in PBMCs of acute brucellosis patients was observed in our study (Figure 1E) [15]. In contrast, there was a reduction in the abundance of CD4^+^ T cells in acute and sub‐acute patients (Figure 1E). This aligns with earlier reports which observed a significant decrease in CD3^+^CD4^+^ T lymphocyte levels in individuals with brucellosis when compared to healthy donors [15]. Innate immune cell subsets (e.g., monocytes, MAIT, and γδ T cells) were higher in acute and sub‐acute patients but decreased in chronic patients. This is consistent with an earlier study that documented the increase in monocytes in peripheral immune cells of brucellosis patients [16]. Interestingly, previous reports have also provided evidence supporting the alterations on the proportions of peripheral immune cells uncovered in our study [10, 15, 16, 34]. This further validates our scRNA‐seq analysis that *Brucella* infection led to shifts in the proportions of different immune cell types. Overall, *Brucella* infection had an impact on the proportions of different immune cell types.

Inflammation is a characteristic feature of brucellosis [34] yet a comprehensive and systemic investigation into the inflammatory response in brucellosis is still lacking. Hence, we endeavored to explore the potential sources of cytokine production in brucellosis. Our results indicated that the inflammation levels in all brucellosis patients were significantly higher than in healthy donors (Figure 3), with the highest inflammatory state observed in acute patients. Hence, acute brucellosis patients may result in life‐threatening complications, which can include cytokine storm syndrome. This is in line with earlier studies that report zoonotic bacterial infections (such as *Brucella* spp. and *Mycobacterium* spp.) have been associated with the development of an inflammatory cytokine storm [14, 35]. We further determined that the primary source of the cytokine storm in acute patients is primarily due to two distinct cell subtypes: Mono_CD14 and Mono_MDSCs (Figure 3). Although various pro‐inflammatory cytokines (e.g., *TNFSF13B*, *S100A8/A9/A12*, *CXCL2*, *CCL8*, *CXCL8*, and *IL6*) were increased in acute patients, S100A8/A9, mainly released by Mono_CD14 cells, might serve as a central factor in instigating the cytokine storm syndrome (Figure 3). In accordance with an earlier report [14], classical monocytes (Mono_CD14) exhibited significantly higher levels of inflammatory genes from the *S100* family in tuberculosis patients who experienced relatively severe symptoms. A variety of inflammatory cells (such as classical monocytes and granulocytes) displayed overexpression of *S100A8/A9*, and increased serum levels of these molecules have been observed in other infectious diseases (e.g., COVID‐19) [14]. Consistently, we also found that *S100A8/A9* was markedly overexpressed in acute brucellosis patients (Figure 3). Interestingly, the cytokine detection data from plasma supports our scRNA‐seq analysis that acute brucellosis patients exhibit elevated levels of S100A8/A9 complex (Figure 3). The S100A8/A9 complex is known to trigger the pro‐inflammatory response via TLR4‐MyD88 signal pathway [20]. As expected, we identified significant upregulation of genes involved in the TLR4‐MyD88 signaling pathway in acute patients, particularly in inflammatory monocytes (e.g., Mono_CD14 and Mono_MDSCs) (Figure 3, Figure S5). Therefore, blocking the binding of S100A8/A9 to TLR4 may inhibit the downstream pro‐inflammatory signal, making it a promising strategy for designing effective therapeutics against acute brucellosis. The use of anti‐S100A8/A9 treatments in the acute brucellosis phase may modulate the production of these molecules and, in turn, attenuate the cytokine storm syndrome. Furthermore, inflammation in chronic patients did not return to levels observed in healthy donors, suggesting that chronic patients experience a prolonged inflammatory condition.

*Brucella* antigens elicit the production of Th1 cytokines in humans, thus the Th1 immune response is indispensable for eradicating *Brucella* infection [35]. To combat *Brucella* infection, the Th1 immune response results in the secretion of IFN‐γ by antigen‐specific CD4^+^ T‐lymphocytes (CD4_Th1) [35]. This study observed that the levels of IFN‐γ expression in CD4_Th1 cells were notably decreased in chronic patients compared to acute and sub‐acute patients (Figure 2). This result was supported by Procarta cytokine analysis of the plasma (Figure 2) and consistent with previous studies where IFN‐γ was found to be lower in patients with chronic brucellosis [18]. IFN‐γ is important for activating the bactericidal function of macrophages, while also stimulating CD8^+^ T mediated cytotoxicity and potentiating the apoptotic death of infected macrophages. Interestingly, significantly elevated expression of Th1 signatures was also observed in brucellosis patients (Figure 2). However, consistent with IFN‐γ expression, chronic patients exhibited a notable decline in the levels of Th1 signatures in comparison to patients in the acute and sub‐acute phases. Herein, the dysregulated Th1 response, including decreased levels of IFN‐γ and Th1 signature, may be a contributing factor to the compromised immune response against *Brucella* infection in chronic patients.

*Brucella* is capable of parasitizing within human APCs (e.g. mDCs), which can affect various cellular functions, including phagocytosis, phagolysosome fusion, antigen presentation, cytokine secretion, and apoptosis [35]. Our data indicate that APCs displayed a significantly higher phagocytosis and antigen presentation capacity only in those with acute illness compared to healthy controls (Figure 7, Figure S9). Hence, it may be hypothesized that during the initial stages of infection (acute phase), Brucella activates APCs (e.g., mDCs) and initiates Th1 responses. However, in later phases (e.g., sub‐acute and chronic stages), *Brucella* may evade these Th1 responses to establish a chronic infection through different evasion mechanisms, such as downregulating HLA‐II expression in APCs. Consistent with this hypothesis, we did not detect a significant increase in the expression of HLA‐II molecules in mDCs among patients with brucellosis (Figure S9). In addition, we also found that *Brucella* infection may inhibit the apoptosis of APCs in sub‐acute and chronic patients (Figure 7, Figure S9). By manipulating the apoptosis of APCs, *Brucella* can avoid being detected by the host immune system, thereby evading the bactericidal function of immune cells [36]. The findings presented here suggest that *Brucella* may potentially disrupt phagocytosis, antigen presentation, and apoptosis in mDCs, aiding in the establishment of a chronic infection.

Monocytic MDSCs, a specific cluster of myeloid cells featured by decreased *HLA‐II* expression and heightened expression of genes related to neutrophil activation (e.g., *S100A8/A12*), were found to be elevated in acute patients (Figure 2, Figure S2). The expansion of MDSCs is a characteristic response in various inflammatory conditions [37]. As a heterogeneous population of immature monocytes, MDSCs have an important role in suppressing T cells through the expression of inhibitory receptors like PDL‐1 [38]. In addition, the decreased expression of HLA‐DR is a known surrogate marker which indicates monocyte dysfunction and results in decreased responsiveness to microbial stimuli. Thus, we postulate that these MDSCs suppresses the host immune response, potentially exacerbating the pathogenesis of brucellosis, especially for acute patients. Intriguingly, several independent studies in patients with COVID‐19 and tuberculosis have recently been published with similar observations, further bolstering our hypothesis [14, 39]. Hence, these findings from our scRNA‐seq analysis collectively indicate that Monocytic MDSCs may contribute to the suppression of host immune responses in individuals with brucellosis.

Interpretation of this study may be constrained by several important limitations. Our study provides a cross‐sectional view of immune responses at different stages of brucellosis, while it lacks continuous temporal data. The longitudinal studies tracking the same individuals over time would offer more detailed insights into the dynamic changes in immune responses during the course of the infection. In addition, our analysis was confined to peripheral blood mononuclear cells (PBMCs), which may not fully represent the immune landscape within infected tissues. Future studies should include tissue‐specific immune responses to provide a more complete picture of the host‐pathogen interactions.

## CONCLUSION

In summary, our study systematically deciphered the comprehensive immune landscape of peripheral immune cells across different phases of *Brucella* infection, presenting a multitude of immune features for brucellosis that were previously uncharacterized. Our findings revealed significant alterations in immune cell proportions and functions, highlighting the pivotal role of cell‐mediated immunity in combating *Brucella*. The data represents a rich resource for gaining a deeper understanding of immune responses in brucellosis and potentially provides valuable insights for the development of effective therapeutic strategies.

## METHODS

### Ethics approval

Ethics approval for this study was obtained from the Ethics Committee of Beijing Ditan Hospital, Capital Medical University (Ethical approval NO. DTEC‐KY2023‐019‐01) and was performed in accordance with the Declaration of Helsinki for medical research involving human subjects. Written informed consent was acquired from each participant.

### Study design and participants

Twenty‐nine patients diagnosed with brucellosis were recruited and peripheral venous blood samples were collected at the Third People's Hospital of Linfen, Shanxi Province, China in Jun 2023 (Table S1). For the 29 brucellosis cases, the inclusion criteria were: (1) positive culture and/or serological tests according to the Guidelines for the Diagnosis of Human Brucellosis (the National Health Commission of China, WS 269‐2019) [9]; (2) ≥ 18 years old. The exclusion criteria were: (1) being pregnant; (2) having auto‐immune diseases; (3) having malignant tumors; (4) receiving immunosuppressive treatment. For six healthy controls, the inclusion criteria were: (1) no history of Brucellosis; (2) negative seroagglutination test (SAT).

### Classification of brucellosis stages

The classification of Brucellosis stages was based on the Diagnosis of Human Brucellosis (National Health Commission of China, WS 269‐2019) [9]: (1) Acute stage was defined as patients with symptoms of Brucellosis within 3 months, and confirmed by positive serological tests. (2) Sub‐acute stage was defined as patients with symptoms of Brucellosis ranging from 4 to 6 months, and confirmed by positive serological tests. (3) Chronic stage was defined as patients with symptoms of Brucellosis for more than 6 months, and confirmed by positive serological tests. Clinical features and laboratory findings which were used for defining the disease phase are provided in Table S1.

### Single‐cell RNA sequencing and data analysis

Standard density gradient centrifugation was used to isolate PBMCs (peripheral blood mononuclear cells) from fresh blood samples (*n* = 35) [39] with >90% cell viability as confirmed with the Countstar cell viability kit. According to the manufacturer's instructions, the Chromium Single Cell 5′ Kit v2 (10x Genomics; PN‐1000263) was used to prepare the 5′ libraries, and single‐cell RNA sequencing was performed using the Illumina Novaseq. 6000 sequencer (2 × 150 bp).

scRNA‐seq data processing and analysis were performed as previously described [40]. Briefly, a merged filtered gene expression matrix for the 35 samples was generated by employing kallisto/bustools (kb v0.24.4) and the ad. concat function in anndata (ad) (v0.7.6) [39]. Using Scanpy (sc) (v1.9.2), doublets/low‐quality cells were then eliminated, library size normalized to 10,000 reads per cell, and a consensus set of the top 1500 highly‐variable genes (HVGs) with substantial cell‐to‐cell variation were identified [13]. Principal component analysis (PCA) was used to reduce the dimensions to 20 PCA components during data set integration. We then applied the Harmony algorithm for batch effect correction [41] and the Louvain algorithm for unsupervised clustering of the single‐cell data [42].

### Cell clustering and annotations

The sc.tl.louvain function was used to perform two rounds of unsupervised cell clustering based on the neighborhood relations of cells. In the first round of analysis (Louvain resolution of 2.0), we identified nine major cell types: CD4^+^ T cells, CD8^+^ T cells, B cells, MAIT cells, γδ T cells, NK cells, megakaryocytes, monocytes, and dendritic cells. Sub‐clusters within each major cell type, which represent distinct immune cell lineages, were then manually confirmed using canonical marker genes (Table S2). By using the sc.tl.rank_genes_groups function, we then identified cluster‐specific signature genes, which were manually compared to canonical marker genes for cluster annotation (Table S2).

### Identifying changes in immune cell proportion

The proportion of each immune cell type/subtype in various disease conditions was calculated, and their statistical significance was confirmed using a Kruskal–Wallis test with Bonferroni correction. We also used a multivariate ANOVA to investigate the impact of different disease stages and their potential interactions on the proportion of each cell type/subtype [14]. Using the R_O/E_ ratio (the ratio of observed vs randomly expected cell numbers), we further calculated the disease preference for each cell type/subtype, providing insights into their association with specific disease phases [14].

### Determining cell state scores

Pre‐defined gene sets were used to compare the overall activation level or physiological activity of different cell types/subtypes. The gene sets associated with pro‐inflammatory cytokines and inflammatory responses were obtained from published literature (Table S3) [39]. Likewise, the gene sets related to Th1 signatures, naïve state, exhaustion state, cytotoxic state, regulatory effector, and IFN‐ response were collected from previous reports (Table S3) [14]. Using the sc.tl.score_genes function, we determined the cell state score, which was defined as the average gene expression of the predefined gene set divided by the reference genes. A Kruskal–Wallis test with Bonferroni correction was performed to test the statistical significance of each cell state score when comparing different disease phases.

### Plasma cytokine assays

Plasma cytokine levels were measured using the Th1/Th2 34‐plex human ProcartaPlex kit (Thermo Fisher Scientific) following manufacturer's instructions and methods described in previous reports [43].

### Statistical analysis

All statistical analysis and data visualization were performed using Python and R. In every figure, we employed the following symbols to represent statistical significance: ns (*p* > 0.05); *p ≤ 0.05; **p ≤ 0.01; ***p ≤ 0.001; ****p ≤ 0.0001.

## AUTHOR CONTRIBUTIONS

Yi Wang conceived the study. Rong‐Meng Jiang, Ling‐Hang Wang, Xiao Lv, Song‐Nian Hu, and Yi Wang designed the study. Rong‐Meng Jiang, Ling‐Hang Wang, Song‐Nian Hu, and Yi Wang supervised this project. Si‐Yuan Yang, Bing Han, Xiu‐Fang Du, Hua‐Li Sun, Yu‐Feng Du, Yin‐Li Liu, Pan‐Pan Lu, and Jin‐Yu Di performed the experiments. Yi Wang and Rong‐Meng Jiang founded the study and contributed the reagents and materials. Yi Wang contributed to the analysis tools. Yi Wang performed the software. Yi Wang, Laurence‐Don‐Wai Luu, Song‐Nian Hu, Si‐Yuan Yang, Bing Han, and Xiu‐Fang Du analyze the data. Yi Wang drafted the original paper. Yi Wang, Song‐Nian Hu, and Laurence‐Don‐Wai Luu revised and edited this paper. Yi Wang, Laurence‐Don‐Wai Luu, Song‐Nian Hu, Ling‐Hang Wang, and Rong‐Meng Jiang reviewed the paper. All authors have read and agreed to the published version of the manuscript.

## CONFLICT OF INTEREST STATEMENT

The authors declare no conflict of interest.

## ETHICS STATEMENT

The ethical approval for this study was obtained from the Ethics Committee of Beijing Ditan Hospital, Capital Medical University (Ethical approval no. DTEC‐KY2023‐019‐01). Written informed consent was acquired from each participant.

## Supporting information


**Figure S1:** Detailed data output and visualization of single‐cell transcriptional profiling of PBMCs from 35 subjects, related to Figure 1.
**Figure S2:** Basic characteristics of selected markers for cell sets/subsets in different cell lineages, related to Figure 1.
**Figure S3:** Comparison of different immune cell types among patient groups, related to Figure 2.
**Figure S4:** Identification of hyper‐inflammatory subtypes associated with potential cytokine storm in PBMCs, related to Figure 3.
**Figure S5:** Details of hyper‐inflammatory subtypes associated with potential cytokine storm in PBMCs, related to Figure 3.
**Figure S6:** Characterization of gene expression differences in CD4^+^ T cells across conditions, related to Figure 4.
**Figure S7:** Characterization of gene expression differences in CD8^+^ T cells across conditions, related to Figure 5.
**Figure S8:** Characterization of gene expression differences in NK cells across conditions, related to Figure 6.
**Figure S9:** Characterization of gene expression differences in myeloid cells across conditions, related to Figure 7.
**Figure S10:** Characterization of gene expression differences in monocytes across conditions, related to Figure 7.

**Table S1:** The laboratory findings and clinical features of enrolled brucellosis patients.
**Table S2:** Marker genes and signature genes for monocytes cell subtypes, related to Figure 1, Figure 2, Figure S1 and Figure S2.
**Table S3:** Signature genes used to define functional status in immune cells.
**Table S4:** The DEGs list of CD4^+^ T cells related to Figure 4.
**Table S5:** The DEGs list of CD8^+^ T cells related to Figure 5.
**Table S6:** The DEGs list of Monocytes cells related to Figure 7.

## Data Availability

The data that support the findings of this study are openly available in the China National Center for Bioinformation at https://ngdc.cncb.ac.cn/omix/release/OMIX006680; reference number OMIX006680. The data reported in this paper have been deposited in the OMIX, China National Center for Bioinformation/Beijing Institute of Genomics, Chinese Academy of Sciences (https://ngdc.cncb.ac.cn/omix; accession no. OMIX006680). Supplementary materials (Figure, tables, graphical abstracts, slides, videos, Chinese translated version, and updated materials) may be found in the online DOI or iMeta Science http://www.imeta.science/.
