# Supplementary material for: Single‐cell landscape revealed immune characteristics associated with disease phases in brucellosis patients
Source: Imeta. 2024 Jul 23;3(4):e226. doi: 10.1002/imt2.226 (PMC11316929; doi:10.1002/imt2.226)
Supplement: Supplementary file 1 — Figure S1: Detailed data output and visualization of single‐cell transcriptional profiling of PBMCs from 35 subjects, related to Figure 1. Figure S2: Basic characteristics of selected markers for cell sets/subsets in different cell lineages, related to Figure 1. Figure S3: Comparison of different immune cell types among patient groups, related to Figure 2. Figure S4: Identification of hyper‐inflammatory subtypes associated with potential cytokine storm in PBMCs, related to Figure 3. Figure S5: Details of hyper‐inflammatory subtypes associated with potential cytokine storm in PBMCs, related to Figure 3. Figure S6: Characterization of gene expression differences in CD4+ T cells across conditions, related to Figure 4. Figure S7: Characterization of gene expression differences in CD8+ T cells across conditions, related to Figure 5. Figure S8: Characterization of gene expression differences in NK cells across conditions, related to Figure 6. Figure S9: Characterization of gene expression differences in myeloid cells across conditions, related to Figure 7. Figure S10: Characterization of gene expression differences in monocytes across conditions, related to Figure 7. [file IMT2-3-e226-s002.docx]

**Supporting information to**

**Single-cell landscape revealed immune characteristics associated with disease phases in brucellosis patients**

**Running title**: Single-cell immune landscape of Brucellosis

Yi Wang **^1#*^**, Siyuan Yang **^2,3,4,5#^**, Bing Han **^6,7#^**, Xiufang Du **^8#^**, Huali Sun **^9^**, Yufeng Du **^8^**, Yinli Liu **^8^**, Panpan Lu **^8^**, Jinyu Di **^10^**, Laurence Don Wai Luu **^11*^**, Xiao Lv **^10*^**, Songnian Hu **^12, 13*^**, Linghang Wang **^4*^**, Rongmeng Jiang **^4,7*^**

^1^Experimental Research Center, Capital Institute of Pediatrics, Beijing, 100020, China

^2^Beijing Key Laboratory of Emerging Infectious Diseases, Institute of Infectious Diseases, Beijing Ditan Hospital, Capital Medical University, Beijing 100015, China

^3^Beijing Institute of Infectious Diseases，Beijing, 100015, China

^4^National Center for Infectious Diseases, Beijing Ditan Hospital, Capital Medical University, Beijing 100015, China

^5^National Key Laboratory of Intelligent Tracking and Forecasting for Infectious Diseases, Beijing,100015, China

^6^Clinical and Research Center of Infectious Diseases, Beijing Ditan Hospital, Capital Medical University, Beijing,100015, China

^7^Beijing Quality Control and Improvement Center of Infectious Disease, Bejing, 100015, China

^8^The Department of Infectious Diseases, The Third People’s Hospital of Linfen City, Linfen, Shanxi, 041000, China

^9^Department of Infectious Diseases, The Afliated Hospital of Qingdao University, Qingdao, Shandong, 266001, China

^10^Department of Clinical Laboratory, The Third People’s Hospital of Lifen City, Linfen, Shanxi, 041000, China

^11^School of Life Sciences, University of Technology Sydney, Sydney, 207, Australia

^12^State Key Laboratory of Microbial Resources, Institute of Microbiology, Chinese Academy of Sciences, Beijing, 100101, China

^13^University of Chinese Academy of Sciences, Beijing, 101408, China

^#^These authors contributed equally: Yi Wang, Siyuan Yang, Bing Han, Xiufang Du

^*^Correspondence: [wildwolf0101@163.com](mailto:wildwolf0101@163.com) (Yi Wang); [laurence.luu@uts.edu.au](mailto:laurence.luu@uts.edu.au) (Laurence Don Wai Luu); [13903476639@163.com](mailto:13903476639@163.com) (Xiao Lv); [husn@im.ac.cn](mailto:husn@im.ac.cn) (Songnian Hu); [linghang.wang@ccmu.edu.cn](mailto:linghang.wang@ccmu.edu.cn) (Linghang Wang); [13911900791@163.com](mailto:13911900791@163.com) (Rongmeng Jiang)

**
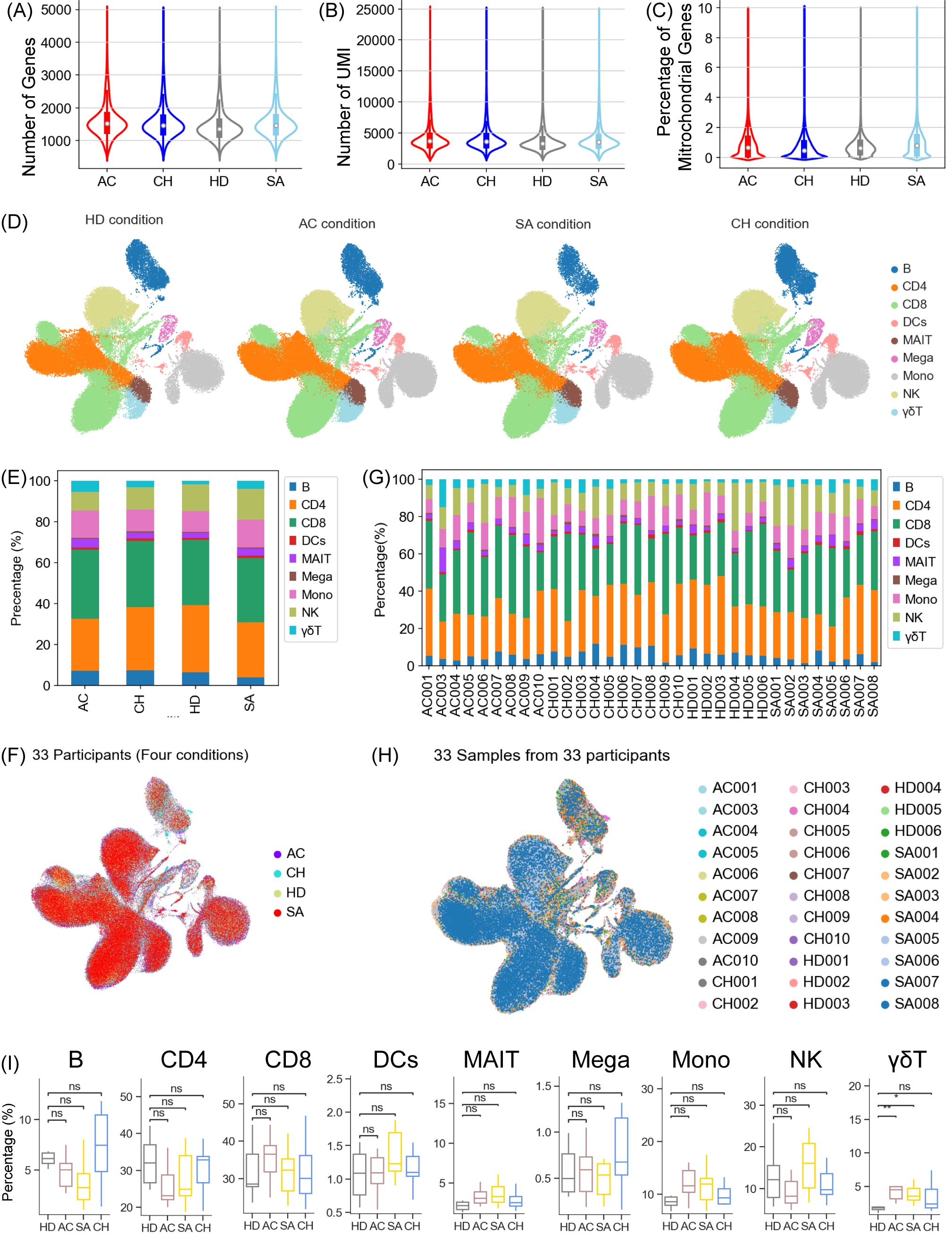
**

**Figure S1** Detailed data output and visualization of single-cell transcriptional profiling of PBMCs from 35 subjects, related to figure 1. (A)-(C). Distribution of the gene counts per cell (A), unique molecular identifier (UMI) counts per cell (B), and percentage of mitochondrial transcripts per cell (C) detected for cells in each group. (D). The UMAP projection for the four conditions on different panels. Cells are colored by the 9 major cell types. (E). Stacked bar plot showing the relative proportion of the 9 cell subtypes derived from AC, SA, CH and HD conditions. (F). The UMAP projection for the four conditions. Cells are colored according to each condition. (G). Stack bar plot showing the relative proportion of the 9 cell subtypes for each of the 35 samples. (H). The UMAP projection for the 35 individual samples. Cells are colored according to each sample. (I). The distribution of each immune cell type across the 4 conditions. The y-axis shows the average percentage of each immune cell type. Conditions are displayed in different colors on the *x* axis.


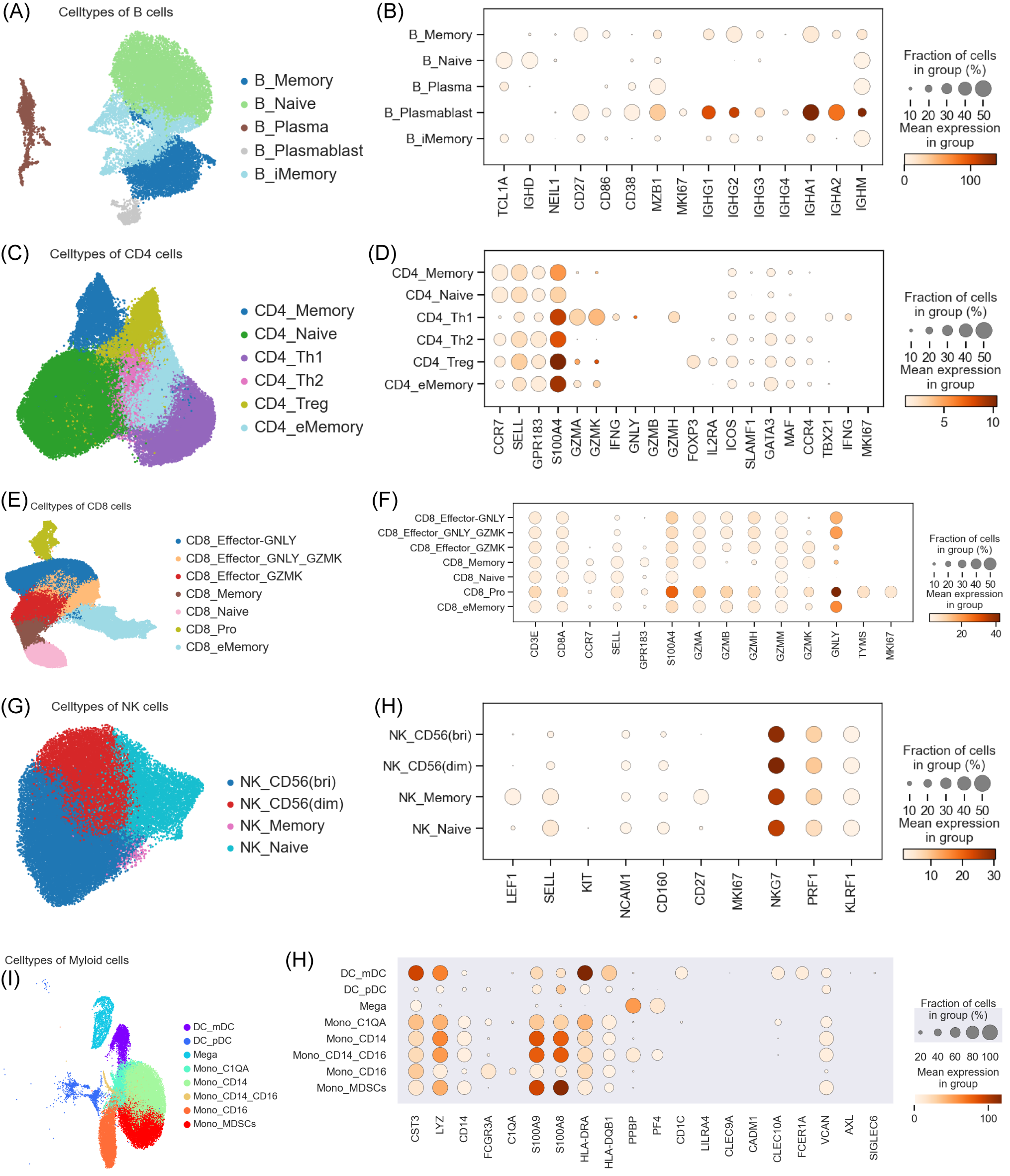


**Figure S2** Basic characteristics of selected markers for cell sets/subsets in different cell lineages, related to Figure 1.

(A), (C), (E), (G) and (I). The clustering result of B (A), CD4^+^T (C), CD8^+^T (E), NK (G) and Myeloid (I) cell subsets. Each point represents one single cell, colored according to cell type. (B), (D), (F), (H), (J). Dot plots of selected marker genes (Rows) for cell subsets (Columns) within each cell lineage, including B (B), CD4^+^T (D), CD8^+^T (F), NK (H) and Myeloid (J) cell subsets.


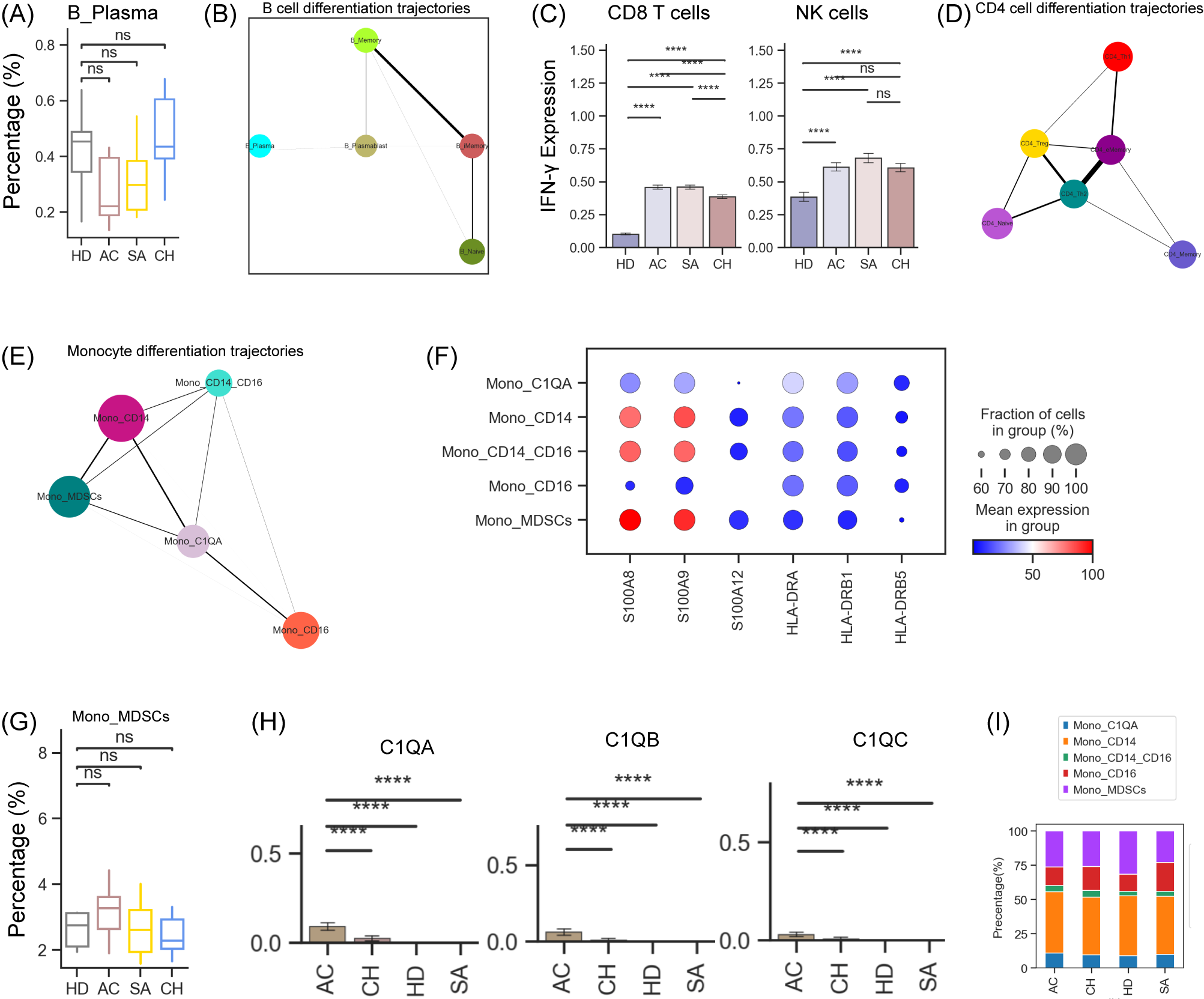


**Figure S3** Comparison of different immune cell types among patient groups, related to Figure 2. (A) . The distribution of B_Plasma across the 4 conditions. (B). PAGA analysis of B cell pseudo-time: the associated cell type and the corresponding status are listed. (C). Bar plots showing the IFN-gamma expression in CD8+T (Left panel) and NK (Right panel) cells across conditions. (D). PAGA analysis of CD4^+^ T cell pseudo-time: the associated cell type and the corresponding status are listed. (E). PAGA analysis of monocyte pseudo-time: the associated cell type and the corresponding status are listed. (F). Dot plots of selected marker genes (Rows) for monocyte subsets (Columns) within each cell lineage. (G). The distribution of MDSCs across 4 conditions. (H). Bar plots of selected genes in monocytes across different conditions. (I). Stacked bar plot showing the relative proportion of the 5 monocyte subtypes derived from AC, SA, CH and HD conditions. Significant differences in a, d, f and h were determined by Kruskal-Wallis test with Bonferroni correction (**p* < 0.05, ***p* < 0.01, ****p* < 0.001, *****p* < 0.0001, ^ns^*p* > 0.05). The error bars represent Standard Error (SE), and the median is shown as horizontal bars.

**
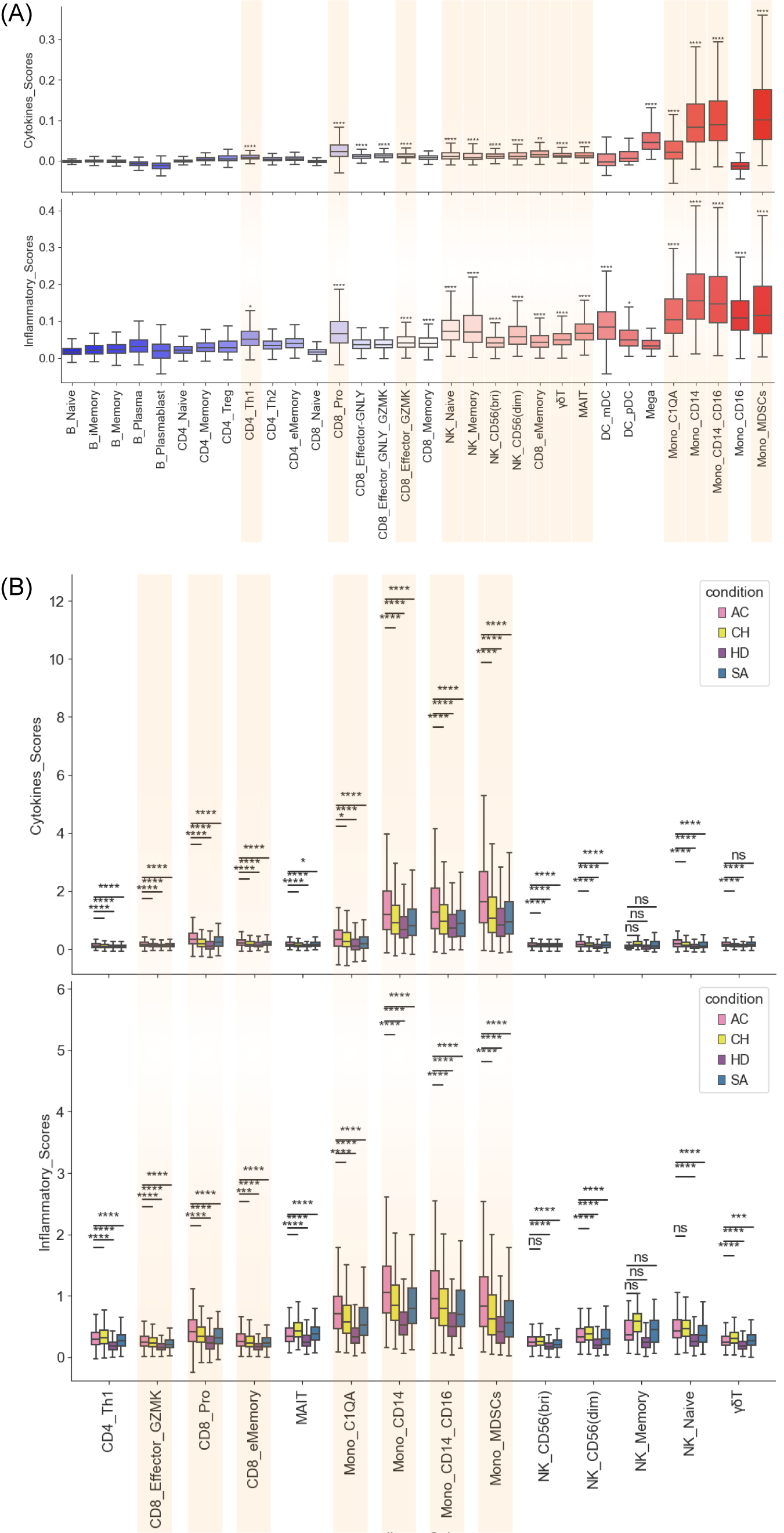
**

**Figure S4** Identification of hyper-inflammatory subtypes associated with potential cytokine storm in PBMCs, related to Figure 3. (A). Boxplots of the inflammatory score (top panel) and cytokine score (bottom panel) for each cell subtype. Significant differences in a, d, f and h were determined by Kruskal-Wallis test with Bonferroni correction (**p* < 0.05, ***p* < 0.01, ****p* < 0.001, *****p* < 0.0001, ^ns^*p* > 0.05). (B). Box plots of the expression levels of inflammatory score (top panel) and cytokine score (bottom panel) derived from healthy donors, acute brucellosis, sub-acute brucellosis and chronic brucellosis. Horizontal lines represent median values, with whiskers extending to the farthest data point within a maximum of 1.5 × interquartile range. Significant differences in a, d, f and h were determined by Kruskal-Wallis test with Bonferroni correction (**p* < 0.05, ***p* < 0.01, ****p* < 0.001, *****p* < 0.0001, ^ns^*p* > 0.05)..

**
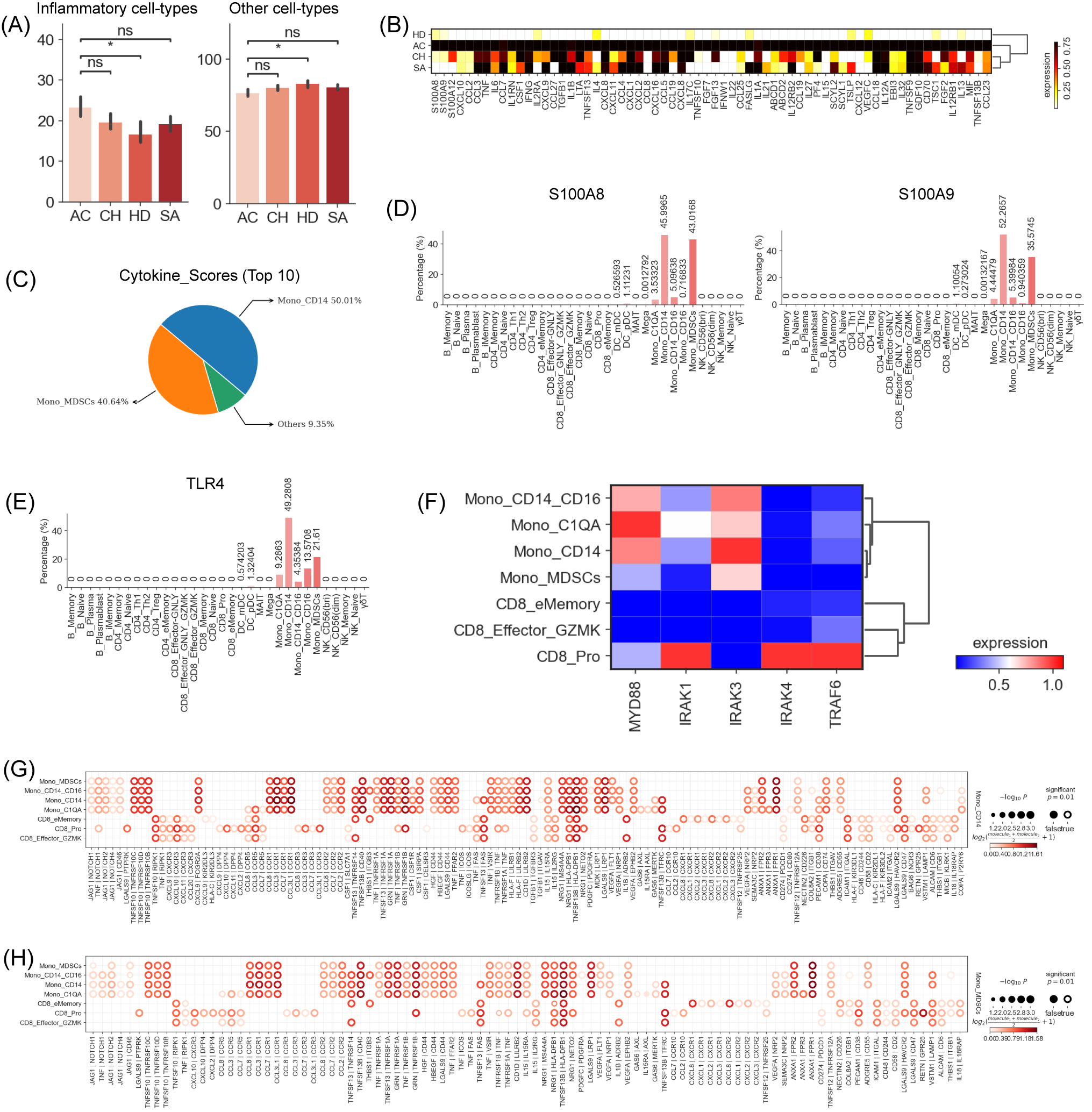
**

**Figure S5** Details of hyper-inflammatory subtypes associated with potential cytokine storm in PBMCs, related to Figure 3. (A). Bar plots showing the proportion of inflammatory cell types (Left panel) and other cell types (Right panel) across conditions. (B). Heatmap depicting the expression of selected pro-inflammatory cytokines across different groups. (C). Pie charts showing the relative percentage contribution of each cell type to the top 10 pro-inflammatory cytokines identified. (D). Bar chart depicting the relative contribution of each cell subtype to *S100A8* and *S100A9* expression. (E). Bar chart depicting the relative contribution of each cell subtype to *TLR4* expression. (F). Heatmap depicting the expression of the selected TLR4-MyD88 signaling genes in the 7 hyper-inflammatory cell subtypes. (G). Circos plot depicting the ligand-receptor pair interactions between Mono_CD14 and the7 hyper-inflammatory cell subtypes. (H). Circos plot depicting the ligand-receptor pair interactions between Mono_MDSCs and the 7 hyper-inflammatory cell subtypes.

**
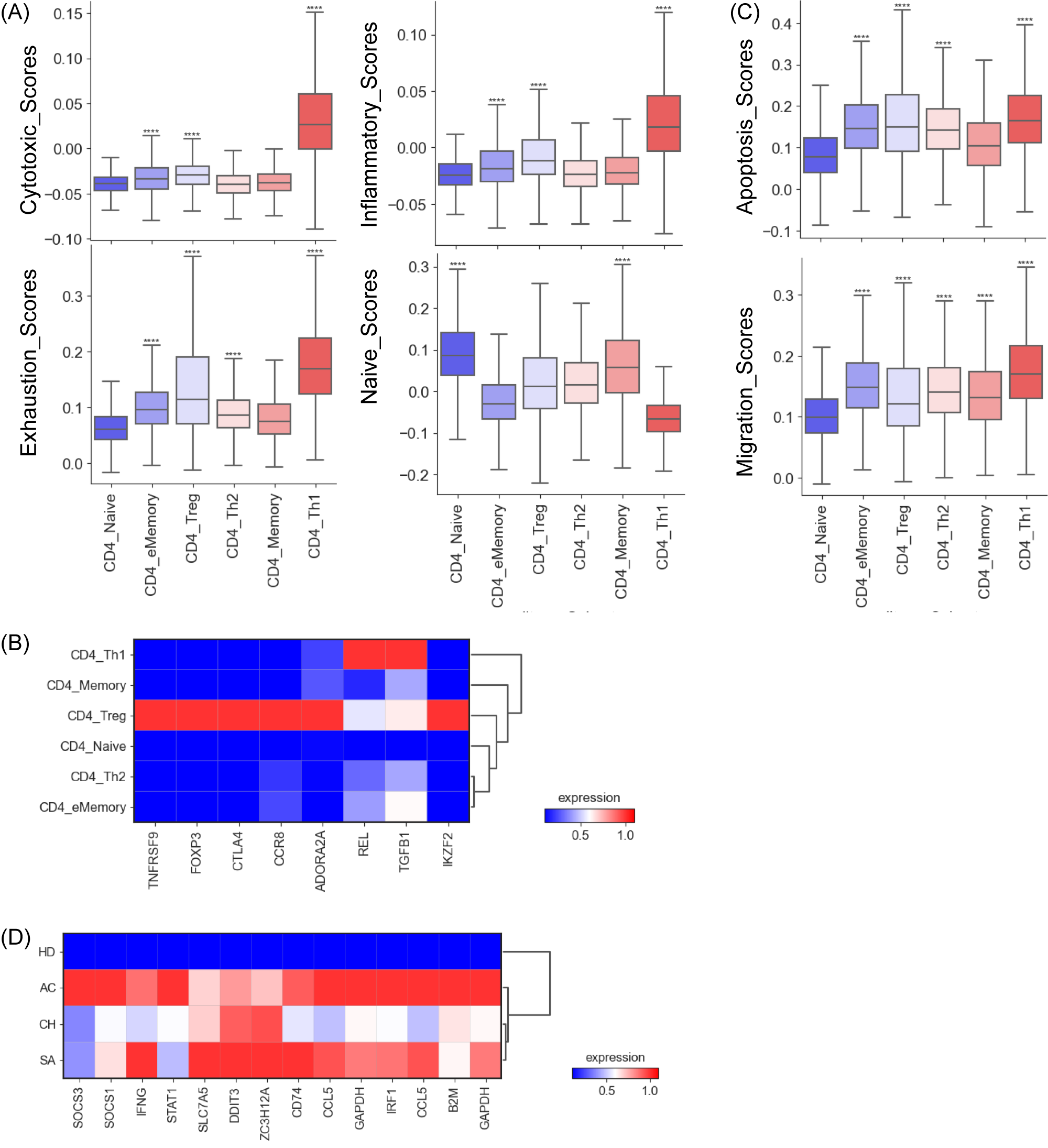
**

**Figure S6** Characterization of gene expression differences in CD4^+^T cells across conditions, related to Figure 4. (A). Box plots showing the naïve, cytotoxic, exhaustion and inflammatory scores for each CD4^+^T cell subsets. (B). Heatmap depicting the expression of selected genes in the 6 CD4^+^T cell subtypes. (C). Box plots showing the apoptosis and migration scores for each CD4^+^T cell subsets. (D). Heatmap depicting the expression of selected genes in different groups.

**
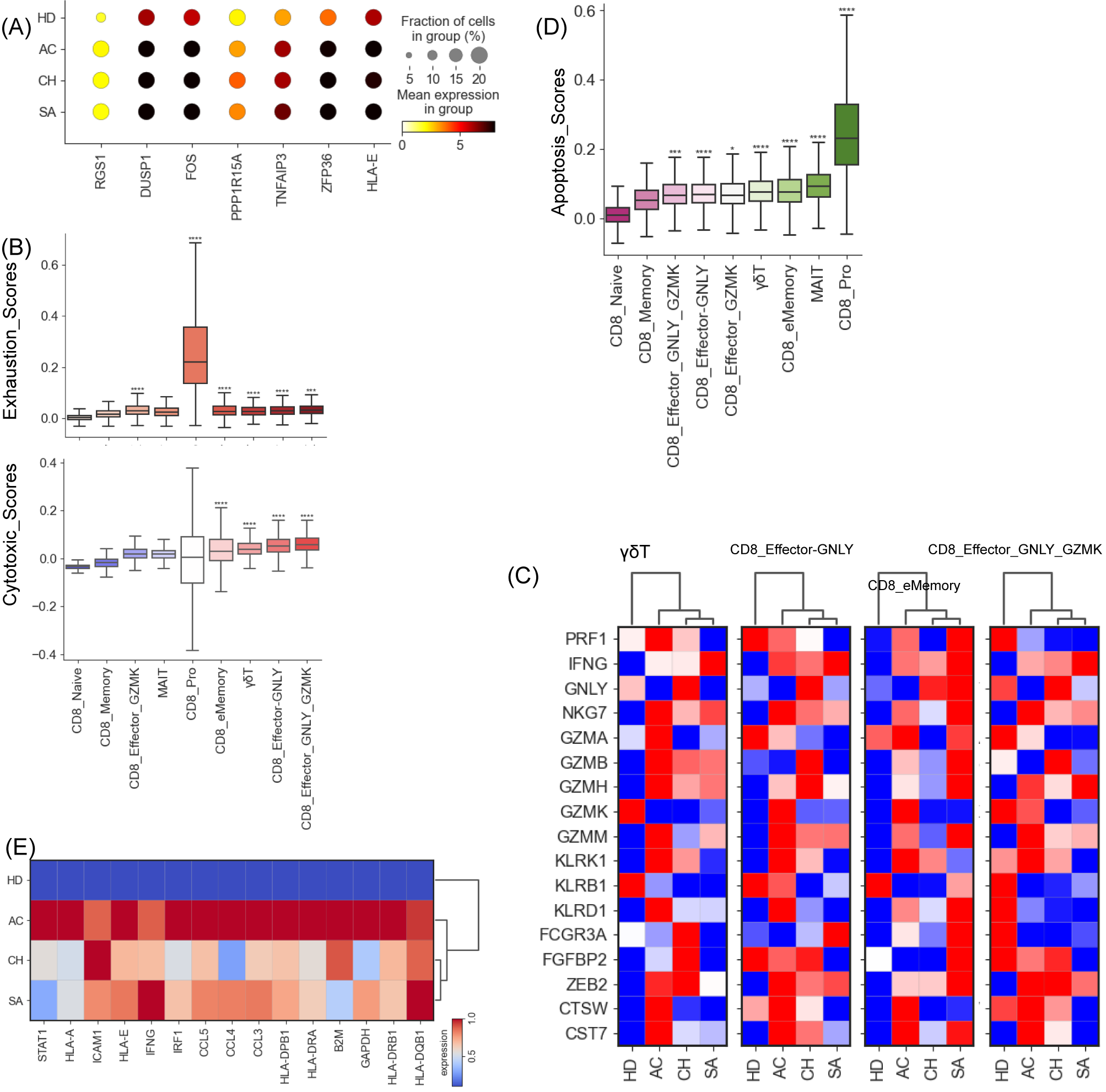
**

**Figure S7** Characterization of gene expression differences in CD8+T cells across conditions, related to Figure 5. (A). Dot plot showing the expression of selected genes in CD8^+^T cells across different groups. (B). Box plots showing the exhaustion and cytotoxic scores of each CD8^+^T cell subsets. (C). Heatmap depicting the expression of selected genes in CD8+T cells across different groups. (D). Box plots showing the Apoptosis score of each CD8^+^T cell subsets. (E). Heatmap depicting the expression of selected cytotoxicity-related genes in effector CD8+T cell subtypes across different groups.

**
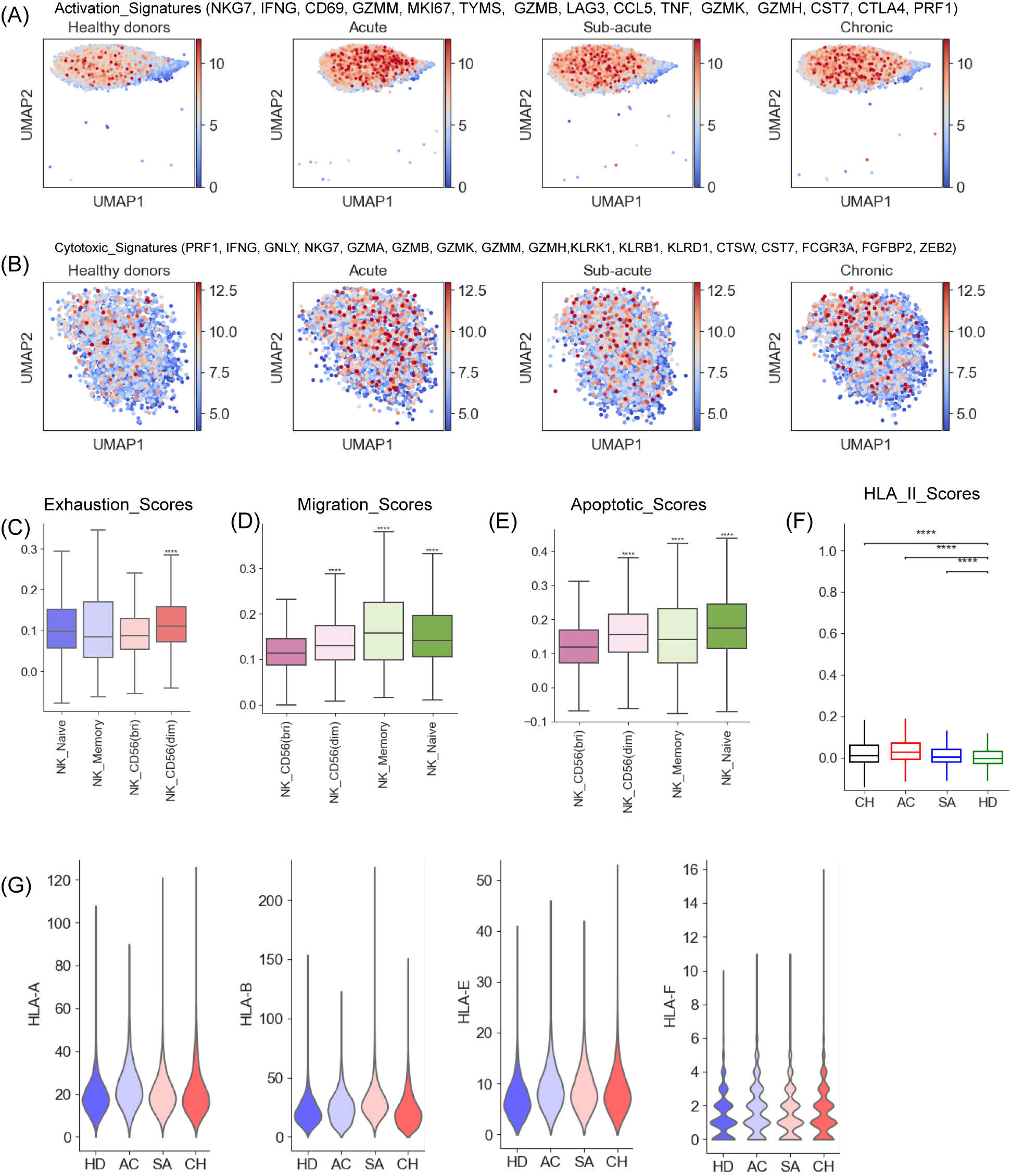
**

**Figure S8** Characterization of gene expression differences in NK cells across conditions, related to Figure 6. (A). UMAP projections for activation signatures in NK cells across different conditions. (B). UMAP projections for cytotoxic signatures in NK_CD56^(dim)^ cells across different conditions. (C)-(E). Box plots showing the Exhaustion score (C), migration scores (D) and apoptosis scores(E)) of each NK cell subsets. (F). Box plots showing the HLA-II Scores of NK cells from different groups. (G). Violin plots showing the expression of HLA-A/B/E/F in NK cells across different conditions.

**
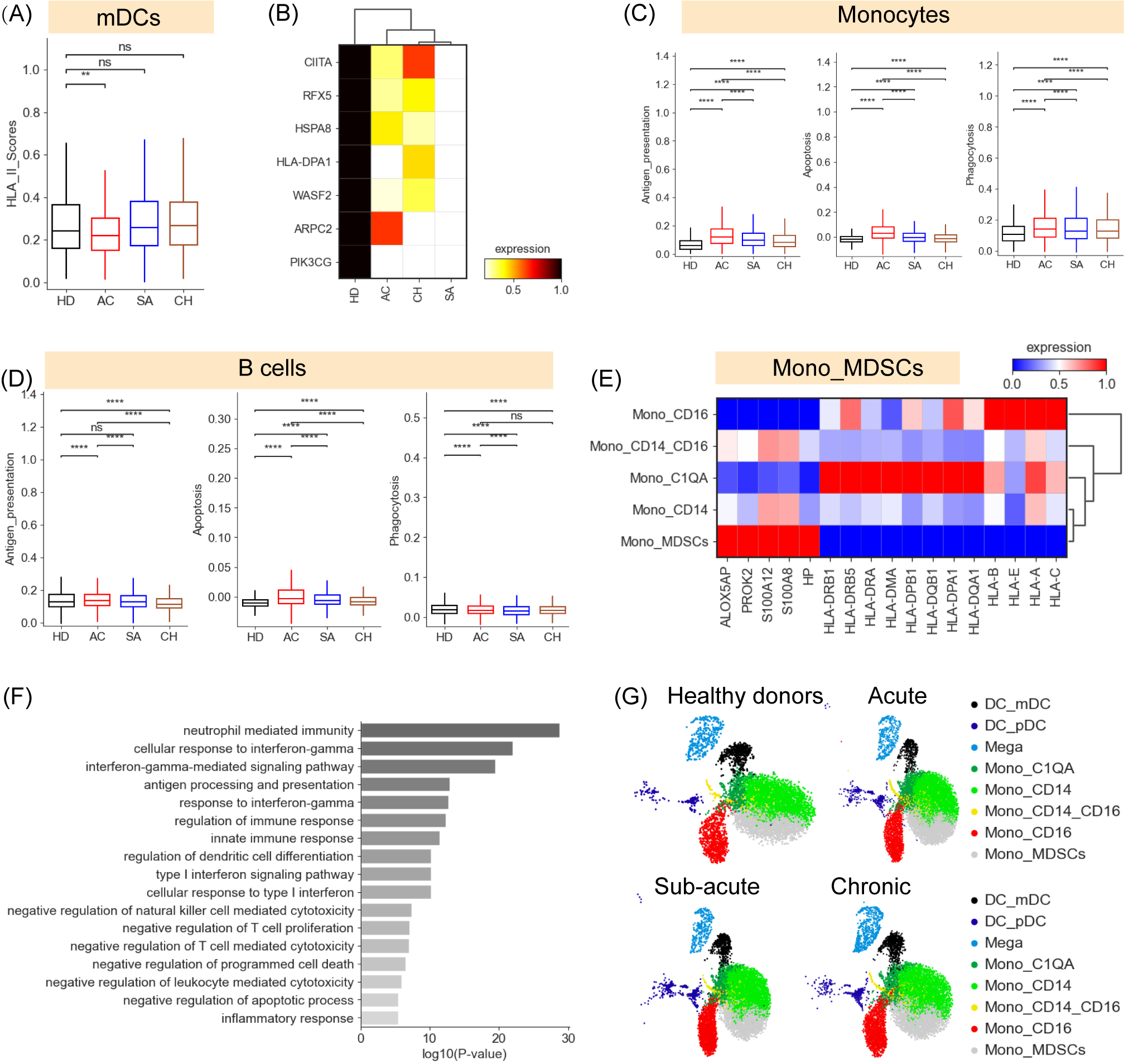
**

**Figure S9** Characterization of gene expression differences in myeloid cells across conditions, related to Figure 7. (A). Box plots showing the HLA-II Scores in mDCs across different groups. (B). Heatmap depicting the expression of antigen presentation-related genes in mDCs across different conditions. (C). Box plot showing the phagocytosis (left), antigen presentation (middle) and apoptosis score (right) in monocytes across different groups. (D). Box plot showing the phagocytosis (left), antigen presentation (middle) and apoptosis score (right) in B cells across different groups. (E). Heatmap depicting the expression of the selected genes in Mono_MDSCs across different groups. (F). Selected enriched GO terms for genes upregulated in Mono_MDSCs. (G). UMAP projection density plots of monocytes from different groups.

**
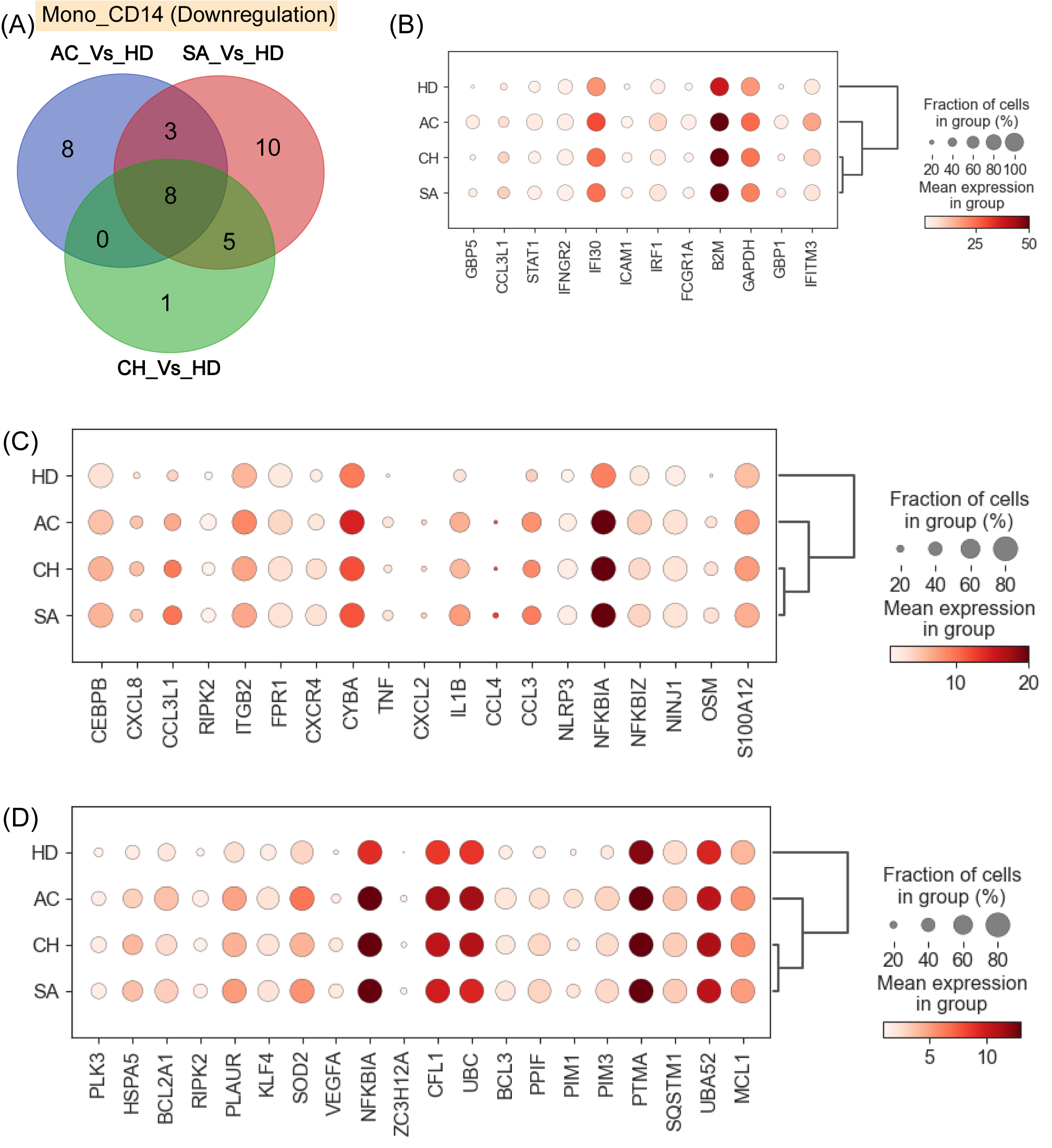
**

**Figure S10** Characterization of gene expression differences in monocytes across conditions, related to Figure 7. (A). Venn diagram illustrating the number of downregulated genes in classical monocytes from different group comparison to health controls. (B). Dot plots showing the expression of IFN-γ/IFN-I response-related genes in classical monocytes across different groups. (C). Dot plots showing the expression of inflammation-related genes in classical monocytes across different groups. (D). Dot plots showing the expression of apoptosis-related genes in classical monocytes across different groups.
